# Equine lentivirus Gag protein degrades mitochondrial antiviral signaling protein via the E3 ubiquitin ligase Smurf1

**DOI:** 10.1128/jvi.01691-24

**Published:** 2024-12-12

**Authors:** Kewei Chen, Bingqian Zhou, Xinhui Wang, Guangpu Yang, Yuezhi Lin, Xuefeng Wang, Cheng Du, Xiaojun Wang

**Affiliations:** 1 State Key Laboratory for Animal Disease Control and Prevention, Harbin Veterinary Research Institute, Chinese Academy of Agricultural Sciences 687216 , Harbin, China; University Hospital Tübingen, Tübingen, Germany

**Keywords:** lentivirus, EIAV, HIV, MAVS, RLR

## Abstract

Equine infectious anemia virus (EIAV) and HIV-1 are both members of the *Lentivirus* genus and are similar in virological characters. EIAV is of great concern in the equine industry. Lentiviruses establish a complex interaction with the host cell to counteract the antiviral responses. There are various pattern recognition receptors in the host, for instance, the cytosolic RNA helicases interact with viral RNA to activate the mitochondrial antiviral signaling protein (MAVS) and subsequent interferon (IFN) response. However, viruses also exploit multiple strategies to resist host immunity by targeting MAVS, but the mechanism by which lentiviruses are able to target MAVS has remained unclear. In this study, we found that EIAV infection induced MAVS degradation, and that EIAV Gag protein recruited the E3 ubiquitin ligase Smurf1 to polyubiquitinate and degrade MAVS. The CARD domain of MAVS and the WW domain of Smurf1 are responsible for the interaction with Gag. EIAV Gag is a precursor polyprotein of the membrane-interacting matrix p15, the capsid p26, and the RNA-binding nucleocapsid proteins p11 and p9. Therefore, we analyzed which protein domain of Gag could interact with MAVS and Smurf1. We found that p15 and p26, but not p11 or p9, target MAVS for degradation. Moreover, we identified the key amino acid residues that support the interactions between p15 or p26 and MAVS or Smurf1. The present study describes a novel role of the EIAV structural protein Gag in targeting MAVS to counteract innate immunity, and reveals the mechanism by which the equine lentivirus can antagonize against MAVS.

**IMPORTANCE:**

Host anti-RNA virus innate immunity relies mainly on the recognition by retinoic acid-inducible gene I (RIG-I) and melanoma differentiation-associated protein 5 (MDA5), and subsequently initiates downstream signaling through interaction with mitochondrial antiviral signaling protein (MAVS). However, viruses have developed various strategies to counteract MAVS-mediated signaling, although the method of antagonism of MAVS by lentiviruses is still unknown. In this article, we demonstrate that the precursor (Pr55gag) polyprotein of EIAV and its protein domains p15 and p26 target MAVS for ubiquitin-mediated degradation through E3 ubiquitin ligase Smurf1. MAVS degradation leads to the inhibition of the downstream IFN-β pathway. This is the first time that lentiviral structural protein has been found to have antagonistic effects on MAVS pathway. Overall, our study reveals a novel mechanism by which equine lentiviruses can evade host innate immunity, and provides insight into potential therapeutic strategies for the control of lentivirus infection.

## INTRODUCTION

The activation of the type I interferon (IFN) pathway is crucial for the control of many viral infections. Following viral invasion, host cells detect the presence of viral RNA using endosomal localized Toll-like receptor (TLR) and cytosolic sensors of the retinoic acid-inducible gene-I (RIG-I)-like receptor (RLR) pathway, RIG-I, and melanoma differentiation-associated gene 5 (MDA5) (1). RIG-I and MDA5 are composed of two N-terminal CARDs, a central DEAD box helicase/ATPase domain, and a C-terminal regulatory domain. Although RIG-I and MDA5 differ in the types of viral RNA that they recognize, they share a common signaling pathway that utilizes the adaptor protein MAVS (mitochondrial antiviral signaling protein, also known as ISP-1/VISA/Cardiff) (2–5). The N-terminal MAVS CARD domain mediates the interaction with RIG-I and MDA5 as well as with downstream effectors; an adjacent proline-rich region also recruits downstream signaling molecules; and the C-terminal transmembrane domain anchors the protein to the mitochondrial membrane (2). MAVS recruits the E3 ligases TRAF3 and TRAF6, facilitating the activation of IFN regulatory factors (IRFs), NF-κB, and the induction of a host antiviral state (6).

However, RNA viruses have evolved strategies to antagonize the type I IFN signaling pathways. MAVS and MAVS signaling are common targets. The cleavage of MAVS by viral protein is one of the most important antagonistic mechanisms. The function of MAVS relies on its mitochondrial localization. Several viral proteins have protease activity and are able to cleave MAVS from the mitochondrial membrane to inhibit downstream signaling. For example, hepatitis C virus (HCV) protease NS3/4A cleaves MAVS at position 508 (cysteine), resulting in the dislocation of the N-terminal fragment of MAVS from the mitochondria (7). The 3C protein of coxsackievirus B and porcine reproductive and respiratory syndrome virus cleaves MAVS at positions 148 (glutamine) and 268 (glutamic acid), respectively (8, 9). Several viruses inhibit MAVS-mediated signaling by degrading MAVS. The hepatitis B virus X protein (HBX) interacts with MAVS, promoting its degradation through Lys136 ubiquitination of the MAVS protein, disrupting innate immunity (10). The Newcastle disease virus (NDV) V protein recruits E3 ubiquitin ligase RNF5 to polyubiquitinate MAVS, leading to its degradation (11). The open reading frame 9b (ORF-9b) of severe acute respiratory syndrome coronaviruses (SARS-CoV) usurps poly(C)-binding protein 2 (PCBP2) and the homologous E6-AP carboxyl terminus (HECT) domain E3 ligase AIP4 to trigger the degradation of MAVS signalosome (12).

The *Lentivirus* family comprises several viruses, including the human immunodeficiency virus-1 (HIV-1), simian immunodeficiency virus (SIV), and equine infectious anemia virus (EIAV). Previous research has shown that RIG-I or MDA5 is able to detect dimeric and monomeric forms of lentiviral RNA, resulting in the activation of the RLR signaling pathway (13, 14). Genomic HIV-1 secondary-structured RNA is also known to induce innate immune responses through the RIG-I-dependent signaling pathway in primary human peripheral blood mononuclear cells (PBMCs) and macrophages (15). Recently, the cytosolic DEAD-box RNA helicase 3 (DDX3), which resembles cytosolic DEAD box helicase RIG-I and MDA5, was shown to function as a pattern recognition receptor (PRR) for HIV-1 to activate the type I IFN pathway, in which MAVS also acts as a central hub for signal transduction (16, 17). In this study, we found the activation of IFN pathway after EIAV infection is also MAVS dependent. The HIV-1 non-structural proteins Nef and Vpu are known to lead to diminished MAVS expression, but the molecular mechanisms remain largely unknown (18). Interestingly, the viral MAVS antagonists identified to date have been, with the exception of rotavirus VP3, mostly non-structural proteins (19). In this study, we report the unexpected discovery that the EIAV Gag, a major EIAV structural protein, is able to mediate MAVS degradation in the proteasome via the E3 ubiquitin ligase Smurf1. Our study reveals a novel mechanism by which an equine lentivirus can evade host innate immunity and provides insight into potential novel therapeutic strategies for the control of lentivirus infection.

## RESULTS

### EIAV infection reduces MAVS expression to attenuate IFN-β level

**Fig 1:**
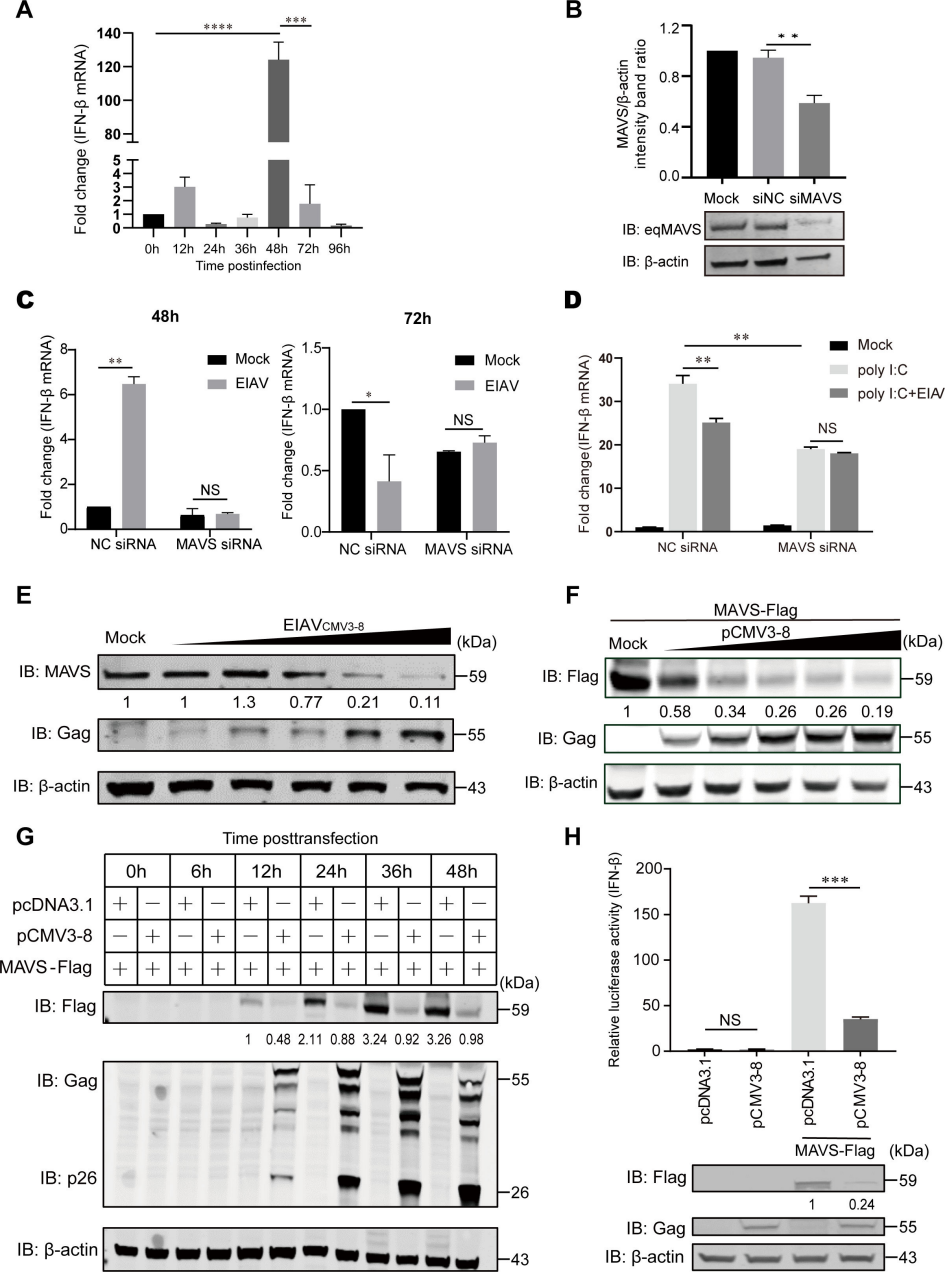
EIAV infection downregulated MAVS expression to inhibit activation of IFN-β. (**A**) The IFN-β mRNA levels in eMDMs infected with EIAV. The fold change in levels of IFN-β mRNA in eMDMs infected with 2 × 10^4^ TCID50 EIAV was measured using qPCR at 0, 12, 24, 36, 48, 72, and 96 hpi. The data represent the means ± SEM from three independent experiments. (**B**) The expression level of MAVS after siRNA knockdown. eMDMs were treated with NC siRNA (siNC) or MAVS siRNA (siMAVS) for 36 h. The cellular lysates were analyzed by immunoblot analysis with anti-MAVS or anti-β-actin. The results of the intensity band ratio of MAVS to β-actin are shown by histogram. The data represent the means ± SEM from three independent experiments. (**C**) The IFN-β mRNA levels in eMDMs were mock treated or infected with EIAV after NC or MAVS siRNA knockdown. The fold change in levels of IFN-β mRNA in NC and MAVS siRNA knockdown eMDMs infected with 2 × 10^4^ TCID50 EIAV was measured using qPCR at 48 and 72 hpi. The data represent the means ± SEM from three independent experiments. (**D**) The mRNA levels of IFN-β in eMDMs (treated by poly I:C) with or without EIAV infection after NC or MAVS siRNA knockdown. The NC and MAVS siRNA knockdown eMDMs were treated with poly I:C (20 µg/mL), then mock infected or infected with 2 × 10^4^ TCID50 EIAV. The fold change in levels of IFN-β mRNA in cell lysate was measured using qPCR at 72 hpi. The data represent the means ± SEM from three independent experiments. (**E**) NBL-6 cells were mock treated or infected with EIAV_CMV3-8_ at a TCID50 of 1 × 10^3^, 5 × 10^3^, 1 × 10^4^, 2 × 10^4^, or 5 × 10^4^ (wedge). Cells were harvested at 72 hpi and were assessed using immunoblot analysis with anti-MAVS, anti-Gag, or anti-β-actin antibody. (**F**) HEK293T cells were mock treated or transfected with pCMV3-8 plasmid at a dose of 0.5, 1, 1.5, 2, or 2.5 µg (wedge), co-transfected with 1 µg MAVS. Cells were harvested at 48 hpt and were assessed using immunoblot analysis with anti-Flag, anti-Gag, or anti-β-actin antibody. For panels E and F, the results of the densitometry analysis to quantify the ratio of MAVS to β-actin are shown at the bottom (lane 1 set as 1). The experiment was performed three times. (**G**) HEK293T cells were co-transfected with 1 µg pCMV3-8 and 1 µg MAVS plasmids. Cells were harvested at 0, 6, 12, 24, 36, and 48 hpt and were assessed using immunoblot analysis with anti-Flag, anti-Gag, anti-p26, or anti-β-actin antibody. The results of the densitometry analysis to quantify the ratio of MAVS to β-actin are shown at the bottom (lane 5 set as 1). This experiment was performed three times. (**H**) HEK293T cells were co-transfected with pGL3-IFN-Luc, PRL-TK, and either an empty vector or pCMV3-8. Simultaneously, cells were mock treated or transfected with 1 µg MAVS-Flag plasmid. Cells were harvested at 24 hpt and were assessed for luciferase activity. The results are presented as relative luciferase activity. Expression levels of expressed proteins were analyzed by immunoblot analysis of the lysates with anti-Flag, anti-Gag, or anti-β-actin antibody. The results of the densitometry analysis to quantify the ratio of MAVS to β-actin are shown at the bottom (lane 3 set as 1). For panels A, B, C, D, and H, significant differences between the different groups were determined using Student’s *t*-tests. *, *P* < 0.05; **, *P* < 0.01; ***, *P* < 0.001. Error bars represent the standard error over three independent experiments.

RLR signaling defends against infections by numerous RNA viruses, including lentiviruses such as HIV-1, through the activation and induction of type I interferons (13–17). Our previous research revealed that, after the infection of the target host cell (equine monocyte-derived macrophages [eMDMs]) with EIAV_FDDV13_, IFN-β expression was upregulated by about fourfold at 12 h and 20-fold at 36 h but reduced to the same level as the control group at 48 h (20). In this study, we also examined dynamic changes in IFN-β expression induced by another virus strain, EIAV_DLV34_. The changing trend of IFN-β expression is similar to previous reports, but the increase of IFN-β expression peaked at 48 h by about 120-fold, and IFN-β expression decreased to about twofold compared with the mock-treated group at 72 h (Fig. 1A). However, to date, there have been no reports addressing the induction of the MAVS signal pathway by EIAV, so MAVS expression was knockdown by siRNA in eMDMs to examine the effect of MAVS in IFN-β induction (Fig. 1B). We found that the EIAV-induced IFN-β pathway is MAVS dependent (Fig. 1C, left). However, previous research has indicated that lentiviruses are able to exploit some strategies to antagonize MAVS signaling, although the exact mechanism is still unclear (13, 18). Our result showed that EIAV (EIAV_DLV34_) infection is able to attenuate IFN-β expression through MAVS in the eMDMs (Fig. 1C, right). Moreover, EIAV (EIAV_DLV34_) infection could inhibit the activation of the MAVS-dependent IFN-β pathway, which is induced by poly I:C, in eMDMs (Fig. 1D). To explore whether EIAV targets MAVS to inhibit IFN-β production, we investigated MAVS expression levels following EIAV infection. The results showed that a significant decrease of endogenous MAVS expression level was detected at 72 h EIAV_CMV3-8_ post-infection (hpi) in a dose-dependent manner (Fig. 1E). To further verify that EIAV is able to reduce MAVS expression, the EIAV_CMV3-8_ infectious clone plasmid pCMV3-8 and MAVS-Flag were co-transfected into HEK293T cells. As expected, the expression of pCMV3-8 was able to reduce MAVS expression at 24 h post-transfection (hpt) in a dose-dependent manner (Fig. 1F). Moreover, the time dynamics results of MAVS protein level after pCMV3-8 transfection showed that MAVS expression is downregulated at 12, 24, 36, and 48 hpt and peaked at 36 hpt (Fig. 1G). The role of EIAV_CMV3-8_ in the downstream activation of IFN-β was then evaluated. HEK293T cells were co-transfected with IFN-Luc, MAVS-Flag, and pCMV3-8 or the empty vector pcDNA3.1. As expected, the IFN-β promoter activity was inhibited after co-transfection of pCMV3-8, compared with that of the empty vector (Fig. 1H). Collectively, our results demonstrate that replication-competent EIAV could reduce MAVS expression.

### EIAV Gag protein reduces MAVS expression level

**Fig 2:**
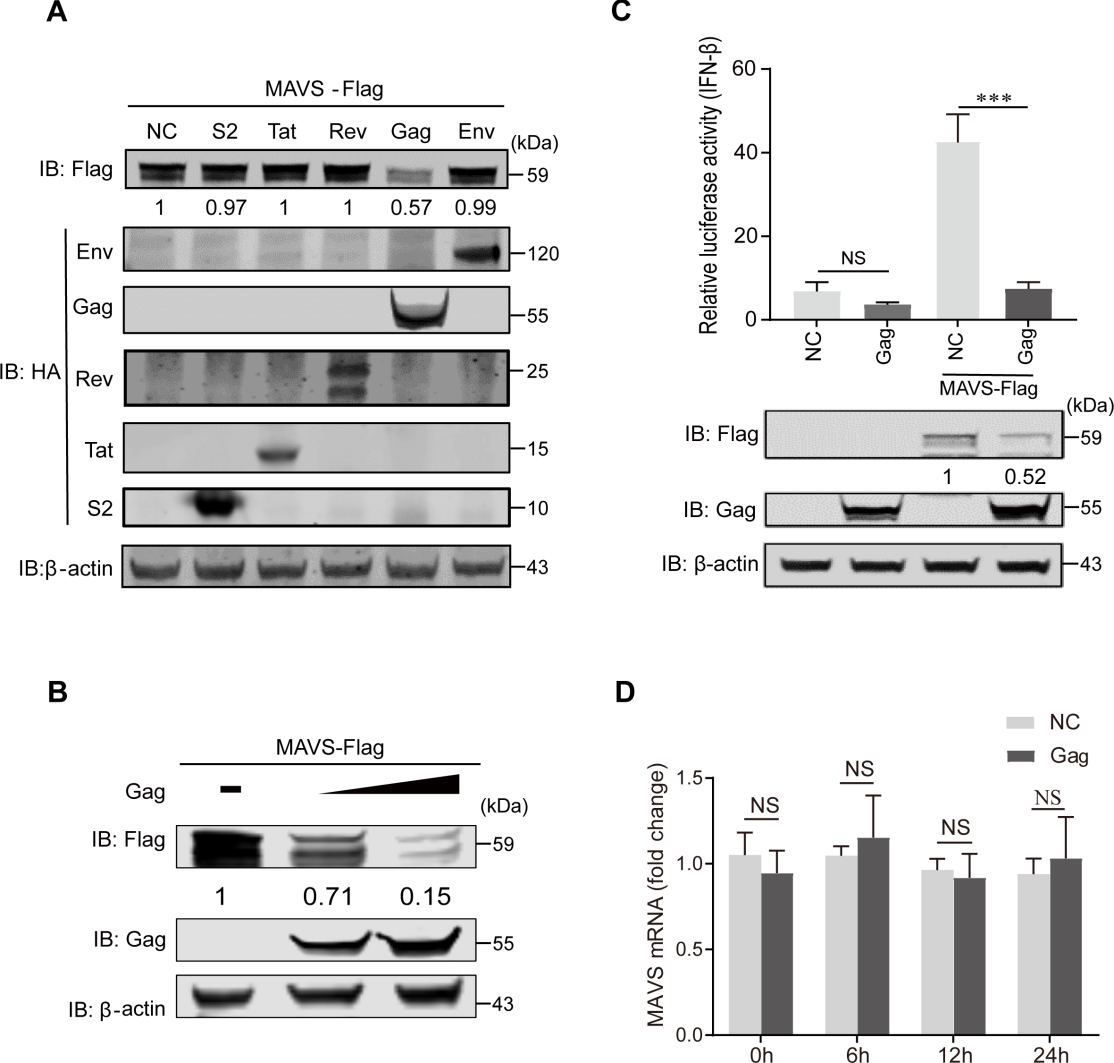
EIAV Gag protein triggers MAVS degradation. (**A**) HEK293T cells were co-transfected with either an empty vector (negative control, NC) or HA-tagged S2, Tat, Rev, Gag, or Env and MAVS-Flag. Cells were harvested at 24 hpt and were assessed using immunoblot analysis with anti-HA, anti-Flag, or anti-β-actin antibody. (**B**) HEK293T cells were co-transfected with either an empty vector or MAVS-Flag and Gag (1 or 2 µg; wedge). Cells were harvested at 24 hpt and were assessed using immunoblot analysis with anti-Flag, anti-Gag, or anti-β-actin antibody. For panels A and B, the results of the densitometry analysis to quantify the ratio of MAVS to β-actin are shown at the bottom (lane 1 set as 1). The experiment was performed three times. (**C**) HEK293T cells were co-transfected with pGL3-IFN-Luc, pRL-TK, and either an empty vector or Gag plasmid. Simultaneously, cells were mock treated or transfected with 1 µg MAVS-Flag plasmid. Cells were harvested at 24 hpt and were assessed for luciferase activity. The results are presented as relative luciferase activity. Expression levels of expressed proteins were analyzed by immunoblot analysis of the lysates with anti-Flag, anti-Gag, or anti-β-actin antibody. The results of the densitometry analysis to quantify the ratio of MAVS to β-actin are shown at the bottom (lane 3 set as 1). (**D**) HEK293T cells were mock treated or transfected with Gag. Cells were harvested at 0, 6, 12, and 24 hpt and were assessed using qPCR with MAVS mRNA. For panels C and D, significant differences between the different groups were determined using Student’s *t*-tests. NS, not significant, *P* > 0.05; *P < 0.05; **P < 0.01; ***P < 0.001. Error bars represent the standard error over three independent experiments.

Because EIAV infection reduced MAVS expression, we next evaluated which viral components were responsible for this reduction. For EIAV, the envelope protein (Env) and Gag protein are the main structural proteins, and Rev, S2, and Tat are the main accessory proteins (21). The immunoblot results showed that the expression level of MAVS decreases following EIAV Gag protein transfection but not following transfection with EIAV Env, Rev, S2, or Tat protein (Fig. 2A). Furthermore, EIAV Gag protein reduced MAVS expression in a dose-dependent manner (Fig. 2B). The role of viral proteins on downstream IFN-β activation was then evaluated. As expected, IFN-β promoter activity was inhibited after co-transfection with Gag-HA, compared with that of co-transfection with the empty vector (Fig. 2C). We next evaluated whether EIAV Gag affects MAVS expression at the transcriptional level. The results indicated that EIAV Gag did not affect the levels of MAVS mRNA (Fig. 2D). These results demonstrated the critical role of EIAV Gag protein in the decrease of MAVS expression level.

### EIAV Gag protein interacts with MAVS

**Fig 3:**
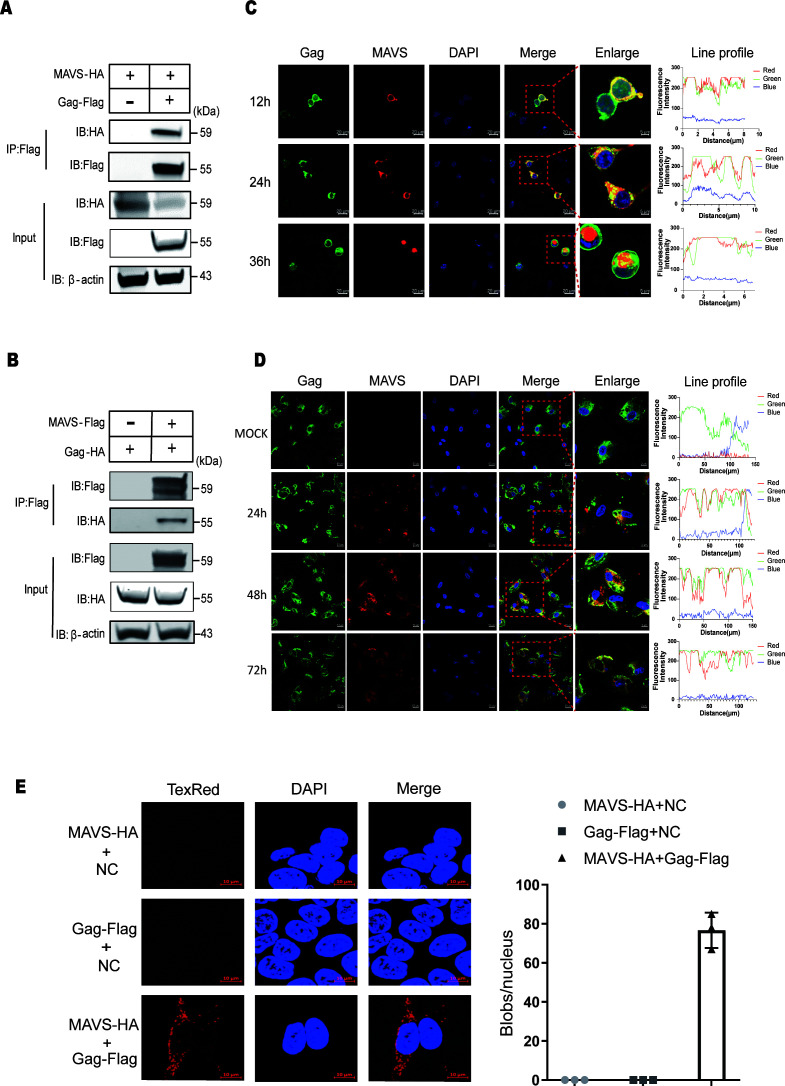
EIAV Gag protein interacts with MAVS in cytoplasm. (**A**) HEK293T cells were co-transfected with MAVS-HA and either an empty vector or Gag-Flag. At 24 hpt, cells were harvested, immunoprecipitated with anti-Flag antibody, and further detected using immunoblot analysis with anti-HA or anti-Flag antibody. (**B**) HEK293T cells were co-transfected with Gag-HA and either an empty vector or MAVS-Flag. At 24 hpt, cells were harvested, immunoprecipitated with anti-Flag antibody, and further detected using immunoblot analysis with anti-HA or anti-Flag antibody. For panels A and B, expression levels of the proteins were analyzed by immunoblot analysis of the lysates with anti-HA, anti-Flag, or anti-β-actin antibody. The experiment was performed three times. (**C**) HEK293T cells were co-transfected with Gag-HA and MAVS-Flag. The cells were fixed at 12, 24, and 36 hpt and were stained with anti-HA or anti-Flag antibody to detect Gag-MAVS (Alexa Fluor 555 [AF555] and AF488 readout) using immunofluorescence assay; DAPI staining (blue) was performed to visualize nuclei (scale bar, 20 µm; 5 µm). Shown is an intensity profile of the linear region of interest (ROI) across the HEK293T cell co-stained with Gag and MAVS. Ten visual fields for each group were examined. (**D**) eMDMs were mock treated or infected with EIAV at 2 × 10^4^ TCID50. Cells were harvested at 24, 48, and 72 hpi and were detected using immunofluorescence assay with anti-MAVS or anti-Gag antibody (Alexa Fluor 555 [AF555] and AF488 readout) (scale bar, 10 µm; 5 µm). Shown is an intensity profile of the linear ROI across the eMDMs co-stained with Gag and MAVS. (**E**) HEK293T cells were co-transfected with Gag-Flag and either an empty vector or MAVS-HA, or MAVS-HA and empty vector. PLA was used to quantify the overlap between MAVS and Gag. The red dots represent the interactions between Gag and MAVS *in situ* (at distances of <40 nm); DAPI staining (blue) was performed to visualize nuclei (scale bar, 10 µm). The number of red dots (blobs/nucleus) is presented by the histogram. The data represent the means ± SEM from 30 cells in three independent experiments.

To investigate a possible interaction between EIAV Gag protein and MAVS, the coimmunoprecipitation of Gag-Flag and MAVS-HA in HEK293T cells were carried out. As shown in Figure 3A, the specific interaction between Gag-Flag and MAVS-HA was detected in the co-transfection group. A reverse immunoprecipitation experiment was performed with Gag-HA and MAVS-Flag, and confirmed the interaction between EIAV Gag and MAVS (Fig. 3B). We then further verified the interaction between EIAV Gag and MAVS by confocal experiment. The results showed that MAVS extensively colocalized with Gag at 12, 24, and 36 hpt (Fig. 3C). To detect whether EIAV Gag was associated with endogenous MAVS in the course of infection, eMDMs were infected with EIAV_CMV3-8_ and were collected at 24, 48, and 72 hpi. Endogenous MAVS was specifically located in the cytoplasm in uninfected or infected cells. The EIAV Gag protein was detected as dispersed fluorescent granules, and some of them were observed to be colocalized with MAVS at 24, 48, and 72 hpi. (Fig. 3D). The Duolink II *in situ* proximity ligation assay (PLA) enables the visualization and quantification of protein interactions (<40 nm) as an individual fluorescent dot by microscopy. We used PLA assay to analyze the interactions between Gag and MAVS. The Gag-Flag and MAVS-HA co-transfection group, but not the control group, had red spots representing interacting complexes (Fig. 3E). Collectively, these results strongly indicated that EIAV Gag was able to interact with MAVS.

### EIAV Gag protein degrades MAVS via E3 ubiquitin ligase Smurf1

**Fig 4:**
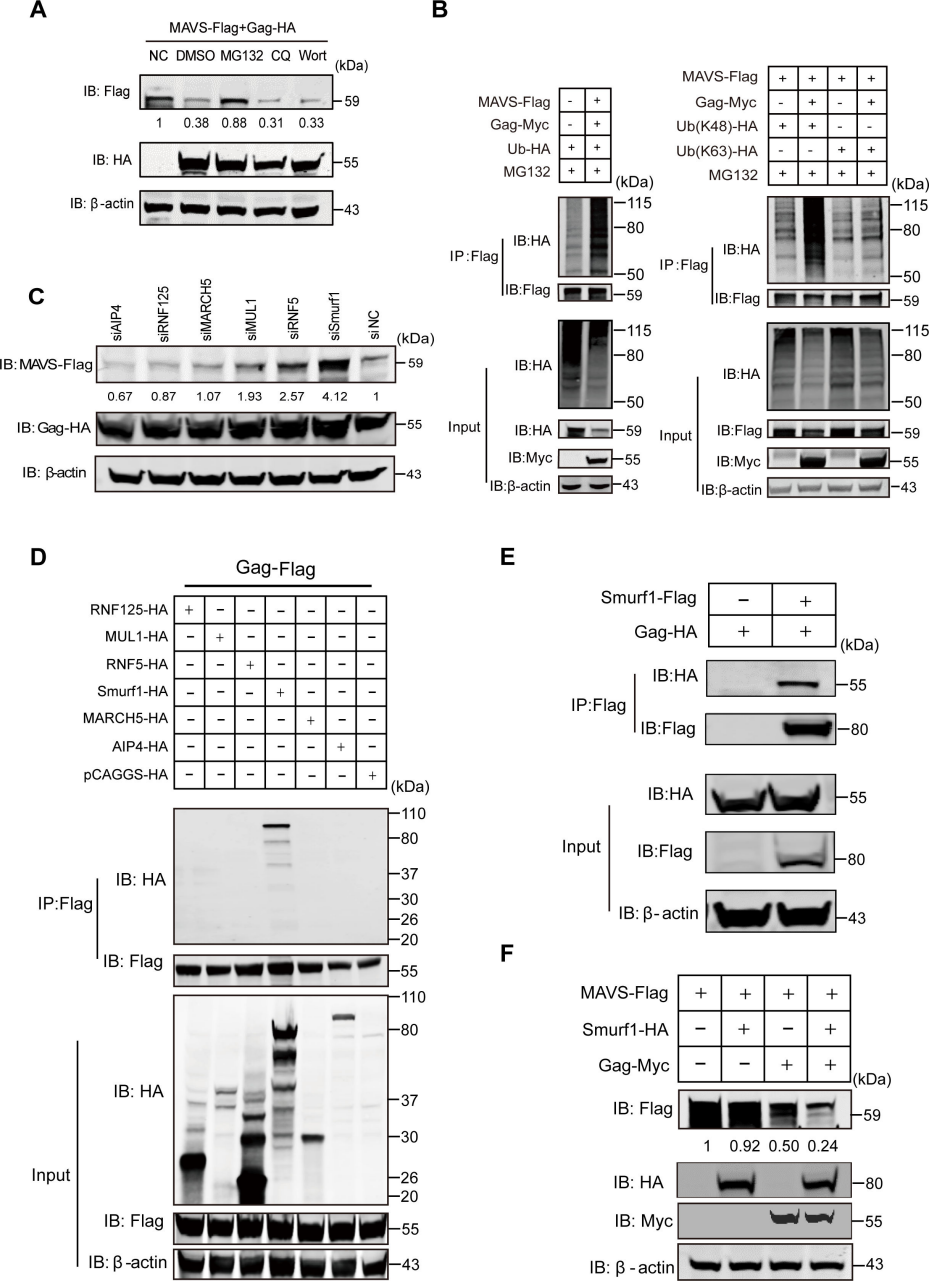
EIAV Gag protein triggers MAVS degradation through Smurf1-mediated ubiquitin-proteasome pathway. (**A**) HEK293T cells were co-transfected with Gag-HA or either an empty vector and MAVS-Flag and were maintained in the presence or absence of the proteasome inhibitor MG132 (20 µM, 6 h prior to immunoblot analysis), the lysosome inhibitor CQ (50 µM), or the autophagy inhibitor wortmannin (100 nM) for 12 h. Cells were harvested and assessed using immunoblot analysis with anti-Flag, anti-HA, or anti-β-actin antibody. The results of the densitometry analysis to quantify the ratio of MAVS to β-actin are shown at the bottom (lane 1 set as 1). (**B**) HEK293T cells were co-transfected with MAVS-Flag, ubiquitin-HA, ubiquitin-HA (K48), or ubiquitin-HA (K63) and either an empty vector or Gag-Myc, and were maintained in the presence of the proteasome inhibitor MG132 (20 µM, 6 h prior to immunoprecipitation). At 24 hpt, cells were harvested, immunoprecipitated with anti-Flag antibody, and further assessed using immunoblot analysis with anti-Flag and anti-HA antibody. Expression levels of the proteins were analyzed using immunoblot analysis of the lysates with anti-Myc, anti-Flag, anti-HA, or anti-β-actin antibody. (**C**) HEK293T cells were transfected with either a scrambled siRNA (siNC) or a specific siRNA targeting AIP4, RNF125, MARCH5, MUL1, RNF5, or Smurf1. At 48 h after transfection, cells were co-transfected with MAVS-Flag and Gag-HA plasmids. Cells were harvested at 18 hpt and were assessed using immunoblot analysis with anti-Flag, anti-HA, or anti-β-actin antibody. The results of the densitometry analysis to quantify the ratio of MAVS to β-actin are shown at the bottom (lane 7 set as 1). (**D**) HEK293T cells were co-transfected with Gag-Flag and HA-tagged RNF125, MUL1, RNF5, Smurf1, MARCH5, AIP4 or the empty vector pCAGGS. (**E**) HEK293T cells were co-transfected with Gag-HA and either an empty vector or Smurf1-Flag. For panels D and E, at 24 hpt, cells were harvested, immunoprecipitated with anti-Flag antibody, and further assessed using immunoblot analysis with anti-HA or anti-Flag antibody. Expression levels of the proteins were analyzed using immunoblot analysis of the lysates with anti-HA, anti-Flag, or anti-β-actin antibody. (**F**) HEK293T cells were co-transfected with MAVS-Flag and either an empty vector or Smurf1-HA or Gag-Myc. Expression levels of the proteins were analyzed using immunoblot analysis of the lysates with anti-HA, anti-Flag, anti-Myc, or anti-β-actin antibody. The results of the densitometry analysis to quantify the ratio of MAVS to β-actin are shown at the bottom (lane 1 set as 1). All experiments were performed three times.

The ubiquitin-proteasome pathway and autophagy-lysosome pathways are major systems to control protein degradation. To investigate which one might play a dominating role in the degradation of MAVS by Gag, we co-transfected Gag-HA and MAVS-Flag into HEK293T cells with or without the proteasome inhibitor MG132 or the autophagy inhibitors chloroquine (CQ) and wortmannin (Wort). We routinely observed a downregulation of MAVS in Gag-overexpressing cells compared with the control; however, this degradation was effectively reversed only by MG132 (Fig. 4A). This indicated that MAVS is degraded by Gag via the ubiquitin-proteasome pathway.

Post-translational modification of proteins via ubiquitination tightly regulates the transmission of the IFN-related RLR signaling pathways. K48-linked and K63-linked polyubiquitination play an indispensable role. We therefore overexpressed ubiquitin-HA or K48-HA or K63-HA ubiquitin plasmid (ubiquitin mutants with single lys48 or lys63 residues) in HEK293T cells for 24 h and then transfected the cells with Gag-Myc and MAVS-Flag. Finally, ubiquitination levels were analyzed. As shown in Figure 4B, EIAV Gag promoted the ubiquitination of MAVS, especially the K48-linked polyubiquitination. It has been extensively shown that protein modified by K48-linked polyubiquitin chains can generally be recognized by the proteasome for degradation through proteasome-dependent pathways.

EIAV Gag does not have the ability to degrade the substrate. A previous report identified Smurf1 as an E3 ubiquitin ligase that mediates MAVS degradation (22). To explore which E3 ubiquitin ligase is involved in MAVS degradation, we designed small interfering RNAs (siRNAs) targeting various E3 ligases and tested their roles in Gag-triggered MAVS degradation. Of these candidate E3 ligases, Smurf1, MUL1, and RNF5 were demonstrated to attenuate Gag-induced MAVS degradation (Fig. 4C). We next cloned the genes encoding Smurf1, MUL1, RNF5, and other E3 ligases, and investigated their abilities to interact with EIAV Gag. We found that only Smurf1 was associated with Gag (Fig. 4D). We performed a reverse immunoprecipitation experiment with Gag-HA and Smurf1-Flag, which further confirmed the interaction between EIAV Gag and Smurf1 (Fig. 4E). Moreover, the immunoblotting result showed that overexpression of Smurf1 could enhance the degradation of MAVS by Gag compared with the control (Fig. 4F). These results demonstrated that the E3 ligase Smurf1 was involved in EIAV Gag-mediated MAVS degradation.

### EIAV Gag protein improves the interaction between MAVS and Smurf1

**Fig 5:**
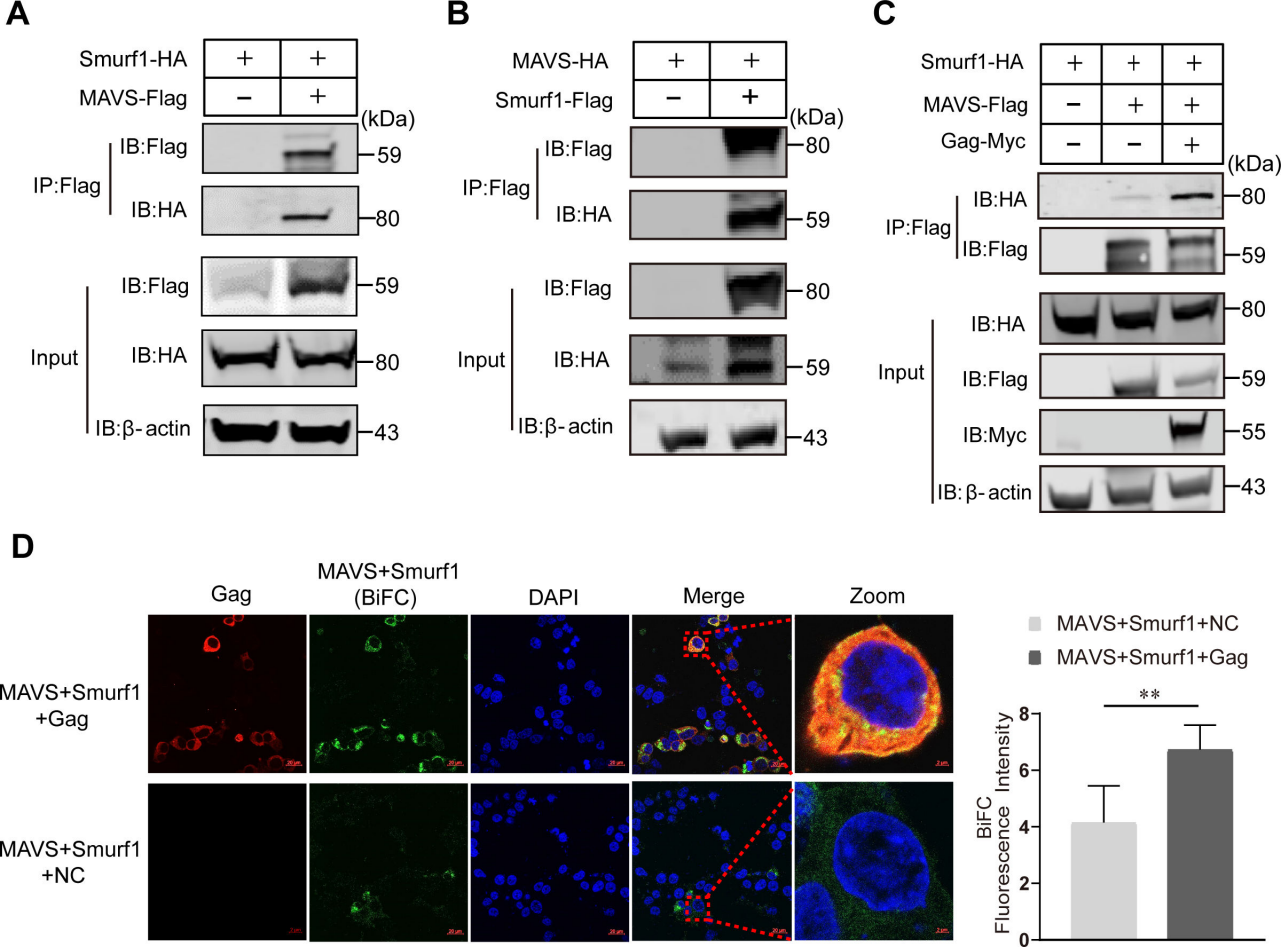
EIAV Gag protein enhances the interaction between MAVS and Smurf1. (**A**) HEK293T cells were co-transfected with Smurf1-HA and either an empty vector or MAVS-Flag. (**B**) HEK293T cells were co-transfected with MAVS-HA and either an empty vector or Smurf1-Flag. (**C**) HEK293T cells were co-transfected with Smurf1-HA and either an empty vector or MAVS-Flag or Gag-Myc. For panels A, B, and C, at 24 hpt, cells were harvested, immunoprecipitated with anti-Flag antibody, and further assessed using immunoblot analysis with anti-HA or anti-Flag antibody. Expression levels of the proteins were analyzed using immunoblot analysis of the lysates with anti-HA, anti-Flag, anti-Myc, or anti-β-actin antibody. All experiments were performed three times. (**D**) Detection of MAVS and Smurf1 interaction using BiFC assay. VN-MAVS and Smurf1-VC were expressed together with or without Gag in HEK293T cells, and Gag protein was stained with mouse anti-Gag antibody followed by Alexa Fluor 647-conjuated rabbit anti-mouse antibody. BiFC green fluorescent signals together with the expression of Gag were visualized using confocal microscopy. DAPI staining (blue) was performed to visualize nuclei (scale bar, 20 µm; 2 µm). The BiFC fluorescence intensity was used to quantify the overlap between MAVS and Smurf1. The fluorescence intensity is presented in the histogram. The data represent the means ± SEM from 30 cells in three independent experiments. Significant differences between the different groups were determined using Student’s *t*-tests. NS, not significant, *P* > 0.05; *P < 0.05; **P < 0.01; ***P < 0.001.

To investigate the molecular mechanism of MAVS degradation by Gag via Smurf1, we analyzed whether MAVS interacts directly with Smurf1, and how Gag is able to affect MAVS/ Smurf1 proteins. To this end, the coimmunoprecipitation of MAVS-Flag and Smurf1-HA in HEK293T cells was carried out. As shown in Figure 5A, a specific interaction between MAVS-Flag and Smurf1-HA was detected in the co-transfection group. A reverse immunoprecipitation experiment performed with Smurf1-Flag and MAVS-HA also confirmed the interaction between MAVS and Smurf1 (Fig. 5B). We then asked whether Gag obviously promoted the co-localization between MAVS and Smurf1 in cells. Our immunoprecipitation results clearly showed that Gag significantly promoted the binding of Smurf1 to MAVS (Fig. 5C). To further confirm the coimmunoprecipitation results, a bimolecular fluorescence complementation (BiFC) assay that could directly visualize protein–protein interactions in living cells (23) was utilized to analyze the specific interaction of MAVS, Smurf1, and Gag. Two constructs were generated: VC-Smurf1-HA, where Smurf1-HA was fused with the C-terminal residues of Venus 174 to 238 (VC), and VN-MAVS-Flag, in which MAVS-Flag was fused with the N-terminal residues 2 to 173 of Venus (VN). After the co-transfection of VC-Smurf1-HA and VN-MAVS-Flag into HEK293T cells, immunostaining allowed a specific BiFC fluorescent signal to be readily detected in the cytoplasmic compartment. Moreover, the BiFC fluorescent signal (green) was enhanced when Gag (red) was co-transfected. Gag was found to be diffused throughout the cytoplasm and almost completely colocalized with the MAVS-Smurf1 BiFC complex (Fig. 5D), which indicated that MAVS, Gag, and Smurf1 can form a complex. Taken together, we think that Gag acts as a trigger factor and promotes the accumulation and binding of Smurf1 protein with MAVS. Smurf1 then promotes the ubiquitination and degradation of MAVS.

### CARD domain of MAVS and WW domain of Smurf1 are responsible for the interaction with Gag

**Fig 6:**
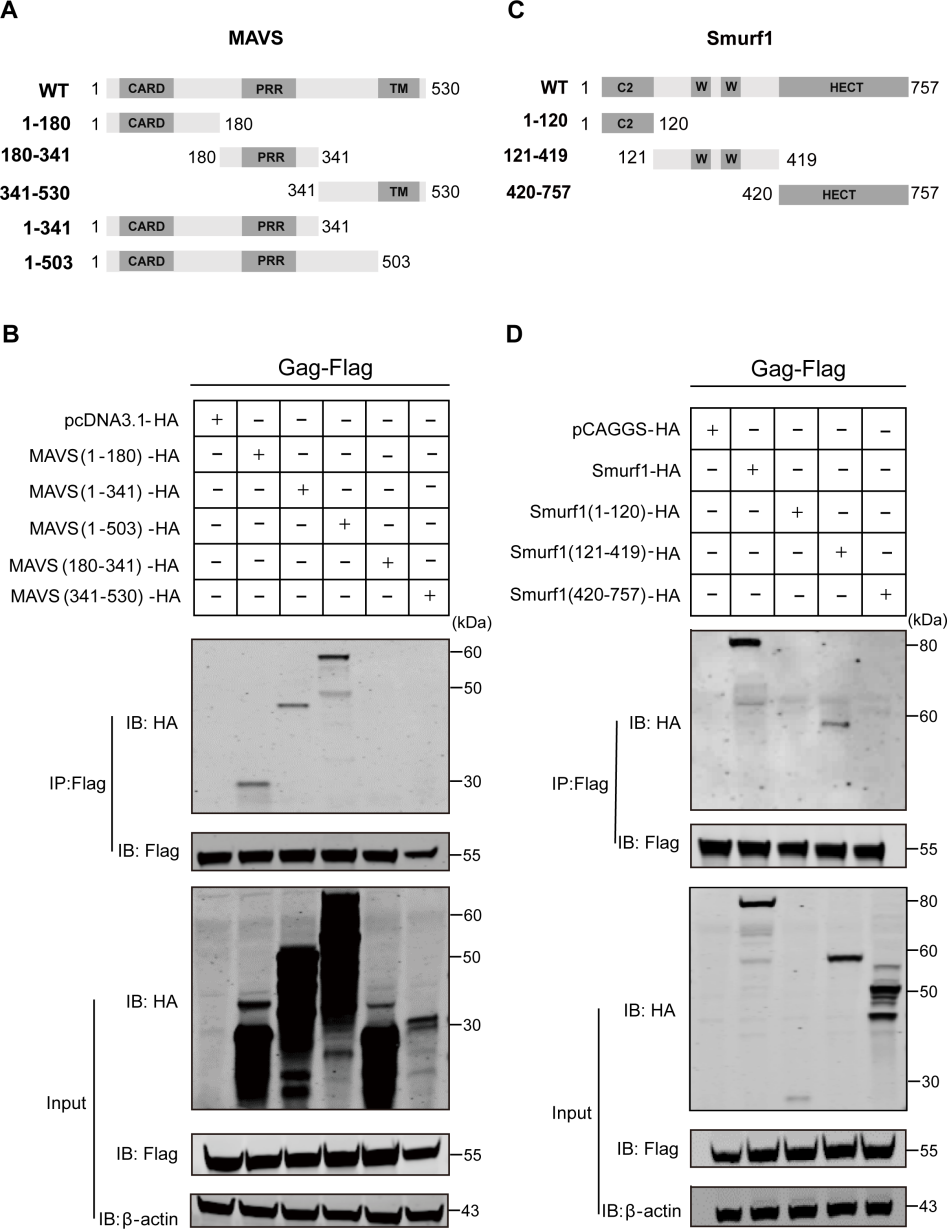
Domain mapping of the Gag-MAVS and Gag-Smurf1 interactions. (**A**) Schematic diagram of wild-type MAVS and structures of respective mutants. (**B**) HEK293T cells were co-transfected with Gag-Flag and either an empty vector or truncated MAVS-HA (aa 1 to 180, aa 1 to 341, aa 1 to 503, aa 180 to 341, and aa 341 to 530). At 24 hpt, cells were harvested, immunoprecipitated with anti-Flag antibody, and further assessed using immunoblot analysis with anti-HA or anti-Flag antibody, and expression levels of the proteins were analyzed using immunoblot analysis of the lysates with anti-HA, anti-Flag, or anti-β-actin antibody. This experiment was performed three times. (**C**) Schematic diagram of wild-type Smurf1 and structures of respective mutants. (**D**) HEK293T cells were co-transfected with Gag-Flag and either an empty vector or wild-type Smurf1 or truncated Smurf1-HA (aa 1 to 120, aa 121 to 419, and aa 420 to 757). At 24 hpt, cells were harvested, immunoprecipitated with anti-Flag antibody, and further assessed using immunoblot analysis with anti-HA or anti-Flag antibody. Expression levels of the proteins were analyzed by immunoblot analysis of the lysates with anti-HA, anti-Flag, or anti-β-actin antibody. This experiment was performed three times.

MAVS contains an N-terminal amino-terminal caspase recruitment domain (CARD), a proline-rich region (PRR) domain, and a C-terminal transmembrane (TM) domain (2) (Fig. 6A). To analyze the critical domain of MAVS responsible for the MAVS–Gag interaction, Gag-Flag was co-transfected with either an empty vector or a truncated MAVS-HA (aa 1 to 180, aa 1 to 341, aa 1 to 503, aa 180 to 341, or aa 341 to 530), and then immunoprecipitation was conducted. We found that the CARD domain of MAVS-HA (aa 1 to 180) had specifically interacted with Gag (Fig. 6B). Deletion of the TM domain did not abrogate MAVS–Gag interaction (Fig. 6B). Therefore, Gag interacted with the aa 1 to 180 of MAVS. Smurf1 comprises an N-terminal C2 domain involved in membrane binding, two central WW domains involved in protein–protein interactions, and a C-terminal HECT (homologous to the E6-AP carboxyl terminus) ubiquitin-ligase domain (Fig. 6C) (24). To determine which domains of Smurf1 are required for Smurf1–Gag interaction, Gag-Flag was co-transfected with either an empty vector, a wild-type (WT) Smurf1-HA, or a truncated Smurf1-HA (aa 1 to 120, aa 121 to 419, aa 420 to 757), and then immunoprecipitation was carried out. The results showed that the WW domain of Smurf1-HA (aa 121 to 419) specifically interacted with Gag (Fig. 6D). Neither the C2 domain nor the HECT domain bound Gag (Fig. 6D). Therefore, Gag interacted with the aa 121 to 419 of Smurf1. In all, we have obtained a domain map of the MAVS-Gag and Smurf1-Gag interaction.

### p15 and p26, but not p11 and p9, of Gag target MAVS for degradation

**Fig 7:**
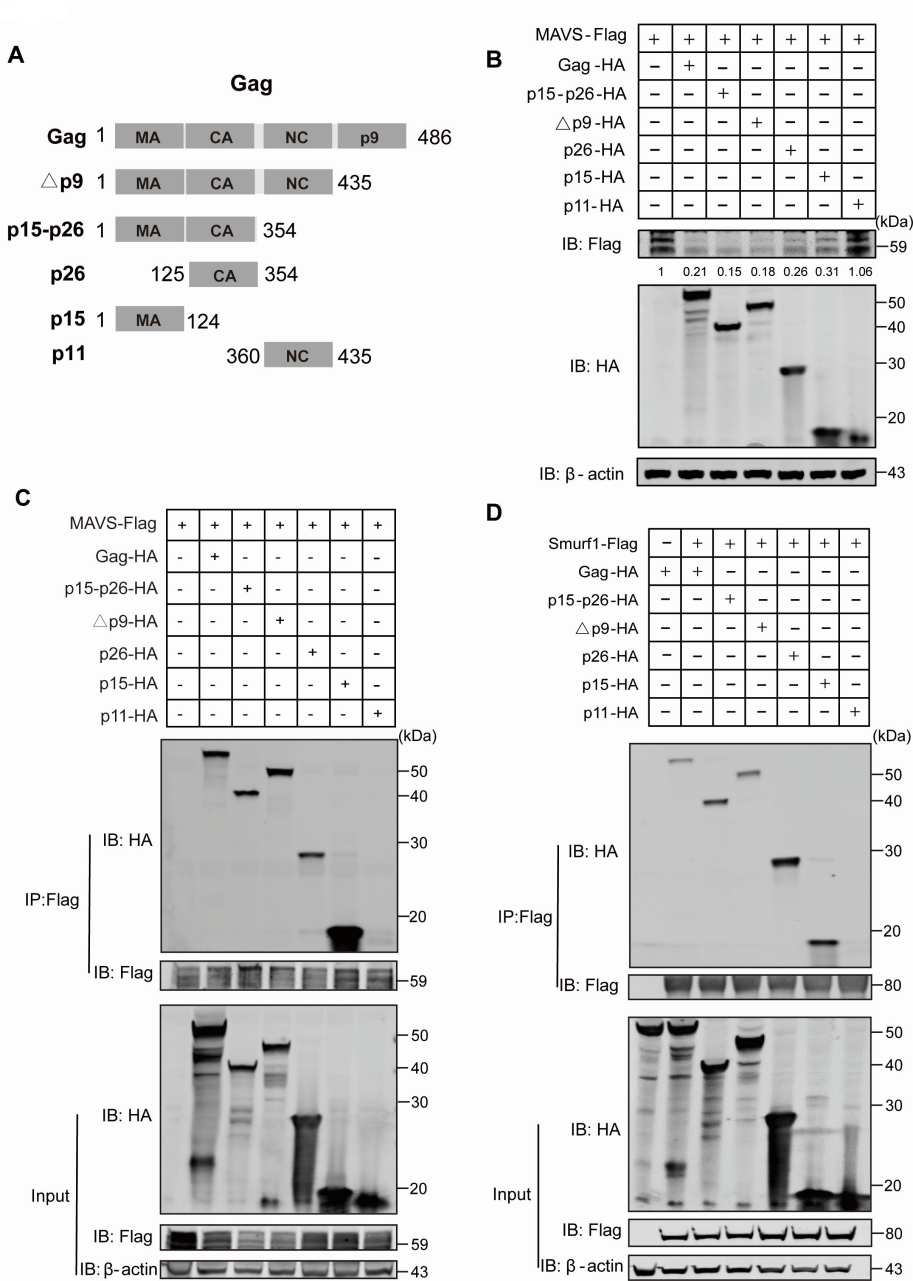
The domains p15 and p26, but not p11 and p9, of Gag could mediate MAVS degradation. (**A**) Wild-type Gag and structures of respective mutants. (**B**) HEK293T cells were co-transfected with MAVS-Flag and either an empty vector or Gag-HA (aa 1 to 486) or Δp9 (aa 1 to 435), p15-p26 (aa 1 to 354), p26 (aa 125 to 354), p15 (aa 1 to 124), and p11 (aa 360 to 435). At 24 hpt, cells were harvested and assessed using immunoblot analysis with anti-Flag, anti-HA, or anti-β-actin antibody. The results of the densitometry analysis to quantify the ratio of MAVS to β-actin are shown at the bottom (lane 1 set as 1). The experiment was performed three times. (**C**) HEK293T cells were co-transfected with MAVS-Flag and either an empty vector or Gag-HA (aa 1 to 486) or Δp9 (aa 1 to 435), p15-p26 (aa 1 to 354), p26 (aa 125 to 354), p15 (aa 1 to 124), and p11 (aa 360 to 435). At 24 hpt, cells were harvested, immunoprecipitated with anti-Flag antibody, and further assessed using immunoblot analysis with anti-HA or anti-Flag antibody. (**D**) HEK293T cells were co-transfected with Smurf1-Flag and either an empty vector or Gag-HA (aa 1 to 486) or Δp9 (aa 1 to 435), p15-p26 (aa 1 to 354), p26 (aa 125 to 354), p15 (aa 1 to 124), and p11 (aa 360 to 435). At 24 hpt, cells were harvested, immunoprecipitated with anti-Flag antibody, and further assessed using immunoblot analysis with anti-HA or anti-Flag antibody. For panels C and D, expression levels of the proteins were analyzed by immunoblot analysis of the lysates with anti-HA, anti-Flag, or anti-β-actin antibody. The experiments were performed three times.

The assembly of Gag precursor proteins on the plasma membrane is essential for virus budding from the host cells. The EIAV Gag-precursor (Pr55gag) polyprotein is cleaved by viral protease into four major internal structural proteins of the mature virion: the membrane-interacting matrix (MA) p15, the capsid (CA) p26, and the RNA-binding nucleocapsid (NC) proteins p11 and p9 (25, 26) (Fig. 7A). After proteolytic processing of Gag, p15 remains associated with the inner face of the viral membrane, and p26 condenses to form a shell around the NC/RNA complex (25–27). We wanted to determine which domain of Gag (p15, p26, p11, and p9) plays a role in the degradation of MAVS. MAVS-Flag was co-transfected with either an empty vector, WT Gag-HA, each of the domains p15-HA, p26-HA, p11-HA, and p15-p26-HA, or a p9 domain deletion mutant (Δp9) of Gag-HA. The immunoblot results showed that deletion of p9 did not abrogate the degradation of MAVS, indicating that p9 is not essential for degradation process. In addition, p15-p26, p15, and p26, but not p11, were able to target MAVS for degradation (Fig. 7B). Further immunoprecipitation results were consistent with the degradation experiment and showed that p15 and p26, but not p11 and p9, were able to bind MAVS or Smurf1 (Fig. 7C and D; Fig. S1). Together, our data suggest Gag mediates proteasome degradation of MAVS through a mechanism dependent on the interaction among MAVS, Smurf1, and p15 or p26.

### The key sites D3/E109 or Q70/R105 in p15 are responsible for their interaction with MAVS or Smurf1

**Fig 8:**
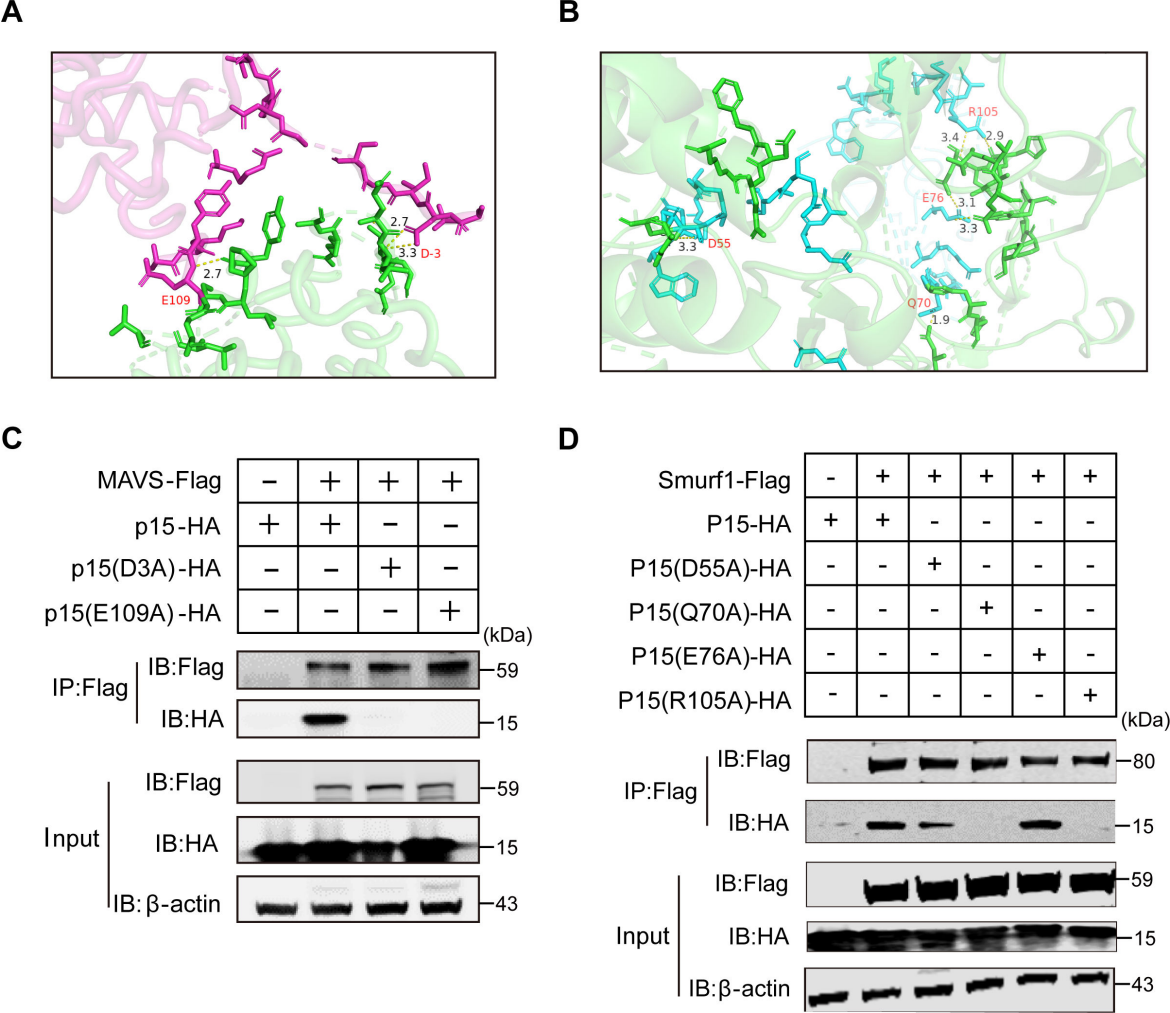
Analysis of key amino acid sites in the MAVS-p15-Smurf1 interactions. (**A**) Structure-based prediction of the interface between p15 (pink) and MAVS (green). Crystal structure of the p15-MAVS conjugate (PDB 1HEK-4O9L) shows non-covalent interactions between p15 and MAVS (possible hydrogen bonds are indicated by yellow dashed lines, with distances in Å). D3 and E109 of p15 facilitate the formation of covalent bonds. (**B**) Structure-based prediction of the interface between p15 (blue) and Smurf1 (green). Crystal structure of the p15-Smurf1 conjugate (PDB 1HEK-F6R1Z0) shows non-covalent interactions between p15 and Smurf1 (possible hydrogen bonds are indicated by yellow dashed lines, with distances in Å). D55, Q70, E76, and R105 of p15 facilitate the formation of covalent bonds. (**C**) HEK293T cells were co-transfected with MAVS-Flag and either an empty vector or p15-HA or the p15(D3A)-HA (from Asp to Ala) and p15(E109A)-HA (from Glu to Ala). At 24 hpt, cells were harvested, immunoprecipitated with anti-Flag antibody, and further assessed using immunoblot analysis with anti-HA or anti-Flag antibody. (**D**) HEK293T cells were co-transfected with Smurf1-Flag and either an empty vector or p15-HA or the p15(D55A)-HA (from Asp to Ala), p15(Q70A)-HA (from Gln to Ala), p15(E76A)-HA (from Glu to Ala), and p15(K105A)-HA (from Lys to Ala). At 24 hpt, cells were harvested, immunoprecipitated with anti-Flag antibody, and further assessed using immunoblot analysis with anti-HA or anti-Flag antibody. For panels C and D, expression levels of the proteins were analyzed using immunoblot analysis of the lysates with anti-HA, anti-Flag, or anti-β-actin antibody. The experiments were performed three times.

Because both p15 and p26 could interact with MAVS or Smurf1, and because these two domains do not have a common functional motif, we asked which amino acid residues were able to support the interactions with MAVS and Smurf1. To answer this question, we firstly performed a simulation analysis on the interaction between p15 and MAVS or Smurf1. The results showed that the residues of D3 and E109, or D55, Q70, E76, and R105 in p15 competitively bound to MAVS or Smurf1 (Fig. 8A or B). To verify these key amino acid sites, we introduced mutations in the corresponding residues in p15. Both the resulting mutants D3A and E109A of p15 showed reduced binding affinity to MAVS (Fig. 8C). Moreover, the resulting mutants Q70A and R105A, but not D55A and E76A, of p15 showed reduced binding affinity to Smurf1 (Fig. 8D). These data suggest that the key sites D3/E109 and Q70/R105 in p15 are responsible for their interaction with MAVS and Smurf1, respectively. Next, we want to know which amino acid residue supports the interactions between p26 and MAVS or Smurf1.

### The key sites Q192/R203 or T16/N83/E115 in p26 are responsible for their interaction with MAVS or Smurf1

**Fig 9:**
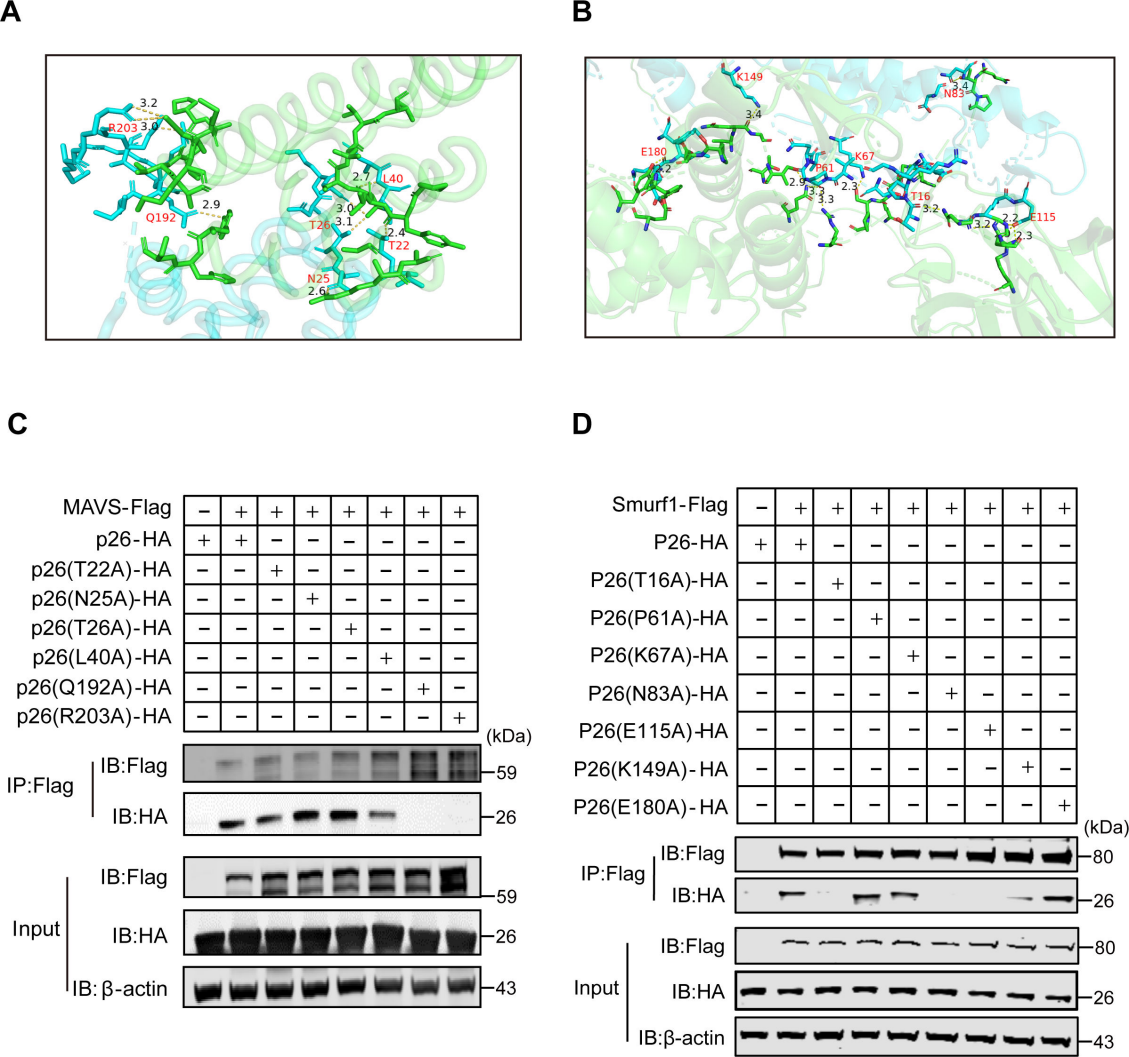
Analysis of key amino acid sites in the MAVS-p26-Smurf1 interactions. (**A**) Structure-based prediction of the interface between p26 (blue) and MAVS (green). Crystal structure of the p26-MAVS conjugate (PDB 1EIA-4O9L) shows non-covalent interactions between p26 and MAVS (possible hydrogen bonds are indicated by yellow dashed lines, with distances in Å). T22, N25, T26, L40, Q192, and R203 of p26 facilitate the formation of covalent bonds. (**B**) Structure-based prediction of the interface between p26 (blue) and Smurf1 (green). Crystal structure of the p26-Smurf1 conjugate (PDB 1EIA-F6R1Z0) shows non-covalent interactions between p26 and Smurf1 (possible hydrogen bonds are indicated by yellow dashed lines, with distances in Å). T16, P61, K67, N83, E115, K149, and E180 of p26 facilitate the formation of covalent bonds. (**C**) HEK293T cells were co-transfected with MAVS-Flag and either an empty vector or p26-HA or the p26(T22A)-HA (from Thr to Ala), p26(N25A)-HA (from Asn to Ala), p26(T26A)-HA (from Thr to Ala), p26(L40A)-HA (from Leu to Ala), p26(Q192A)-HA (from Gln to Ala), and p26(R203A)-HA (from Arg to Ala). At 24 hpt, cells were harvested, immunoprecipitated with anti-Flag antibody, and further assessed using immunoblot analysis with anti-HA or anti-Flag antibody. (**D**) HEK293T cells were co-transfected with Smurf1-Flag and either an empty vector or p26-HA or the p26(T16A)-HA (from Thr to Ala), p26(P61A)-HA (from Pro to Ala), p26(K67A)-HA (from Lys to Ala), p26(N83A)-HA (from Asn to Ala), p26(E115A)-HA (from Glu to Ala), p26(K149A)-HA (from Lys to Ala), and p26(E180A)-HA (from Glu to Ala). At 24 hpt, cells were harvested, immunoprecipitated with anti-Flag antibody, and further assessed using immunoblot analysis with anti-HA or anti-Flag antibody. For panels C and D, expression levels of the proteins were analyzed using immunoblot analysis of the lysates with anti-HA, anti-Flag, or anti-β-actin antibody. The experiments were performed three times.

We also performed a simulation analysis on the interaction between p26 and MAVS or Smurf1. The results showed that the residues of T22, N25, T26, L40, Q192, and R203, or T16, P61, K67, N83, E115, K149, and E180 in p26 competitively bound to MAVS or Smurf1 (Fig. 9A or B). To verify these key amino acid sites, we introduced mutations in the corresponding residues in p26. Both the resulting mutants Q192A and R203A of p26 showed reduced binding affinity to MAVS (Fig. 9C). Moreover, the resulting mutants T16A, N83A, and E115A, but not P61A, K67A, K149A, and E180A, of p26 showed reduced binding affinity to Smurf1 (Fig. 9D). These data suggest that the key sites Q192/R203 and T16/N83/E115 in p26 are responsible for their interaction with MAVS and Smurf1, respectively.

## DISCUSSION

Intact MAVS signaling within host cells is crucial for triggering a robust IFN response to pathogen-associated molecular patterns (PAMPs) and is indispensable for sculpting the adaptive immune response to RNA virus infections (28–30). Since its discovery (2–5), MAVS has become established as a central component of antiviral innate immunity and has served as a prototypic molecule in the study of virus–host co-evolution (31). EIAV and other lentiviruses, such as HIV-1, have been demonstrated to induce high levels of IFN-β and have been widely used as models to study IFN signaling pathways (17, 32, 33). However, growing evidence has shown that HIV-1 employs multiple strategies to counteract IFN signaling, although the mechanism of antagonism of MAVS by lentiviruses remains unclear (13, 16). In the present study, we add to the current understanding of how EIAV, a common lentivirus and an important equine pathogen that is sensitive to the antiviral effects of IFN (34), avoids innate immune surveillance. We provided several lines of evidence to demonstrate that EIAV Gag protein targets MAVS degradation and subsequently inhibits RLR signaling. Both EIAV infection and Gag protein transfection lead to the significant degradation of MAVS and disruption of IFN signaling. EIAV Gag protein specifically interacted with MAVS, targeting its ubiquitination and degradation through E3 ligase Smurf1.

Viral RNA is sensed by sensors such as RIG-I, MDA5, and DDX3, of which the latter two play an important role in detecting HIV-1 RNA (13–17). Transfection of HIV-1 genomic RNA activated RIG-I-dependent signaling (13, 15), but the relevance of RIG-I in the context of HIV-1 infection has not been confirmed, whereas MDA5 is the innate immune receptor for intron-containing RNA from the HIV-1 provirus and that MDA5 potentially contributes to chronic inflammation in people living with HIV-1 (14). DDX3 recognizes prematurely aborted HIV-1 RNA produced during transcription initiation of the provirus (16, 35). The mitochondrial antiviral protein MAVS signals downstream of MDA5 and DDX3, serving as a platform for TBK1/IKKE activation, and is therefore able to elicit the antiviral type I IFN and cytokine responses needed to combat HIV-1 infection (14, 35). However, whether EIAV activates type I IFN response in a MAVS-dependent manner is unknown. Here, we found that type I IFN response induced by EIAV infection was attenuated when the expression of MAVS was knocked down by siRNA transfection (Fig. 1C). This indicated that EIAV infection activates antiviral type I IFN and cytokine responses and that these were elicited through MAVS. Moreover, we found that EIAV infection can inhibit the type I IFN response at 72 hpi (Fig. 1A), and these inhibitory responses depend on MAVS (Fig. 1C). Overall, MAVS functions as a “switch” in the immune signal transduction against equine lentivirus and emerges as the central regulation target by viral evasion.

Viruses can escape the host antiviral immune response by promoting the cleavage or degradation of MAVS and by directly interfering with RLR-activated signaling pathway components. Many virus-encoded proteins are proteases and can cleave MAVS independently of proteasomal degradation or apoptosis to inhibit RLR signaling. The first viral protein reported to colocalize with and cleave MAVS at the mitochondria was HCV serine protease NS3/4A. NS3/4A cleaves MAVS at Cys508, which dislodges the N-terminal fragment of MAVS from the mitochondria, reduces downstream signaling, and enables persistent viral infection (7). The small RNA viruses Seneca Valley virus, human rhinovirus C, and coxsackievirus B3 (CVB3) all encode a cysteine protease, 3Cpro, which cleaves MAVS at Gln148 and inhibits its activity (9, 36, 37). CVB3 also produces a second MAVS-cleaving protease, 2Apro, although the specific cleavage site is unclear (38). In addition to the direct cleavage, some viral proteins induce proteasomal degradation of MAVS. For example, the hepatitis B virus (HBV) protein X (HBX) binds to MAVS and promotes its ubiquitination at Lys136, leading to proteasomal degradation (10). ORF-9b, a protein encoded by coronavirus SARS, catalyzes the K48-linked ubiquitination and degradation of the MAVS signalosome (MAVS, TRAF3, and TRAF6) via the PCBP2–AIP4 axis (12). Interestingly, the NSP1 protein of RVs has E3 ubiquitin ligase-like activity and can target proteins for ubiquitination (39). For example, NSP1 can bind to MAVS CARD or TM domain to promote ubiquitin-dependent proteasomal degradation (40). All the above viral proteins with antagonistic MAVS activity were non-structural proteins. However, in this study, we found that the EIAV Gag, which is a major EIAV structural protein, mediates MAVS degradation via the proteasome (Fig. 2A and 4A).

**Fig 10:**
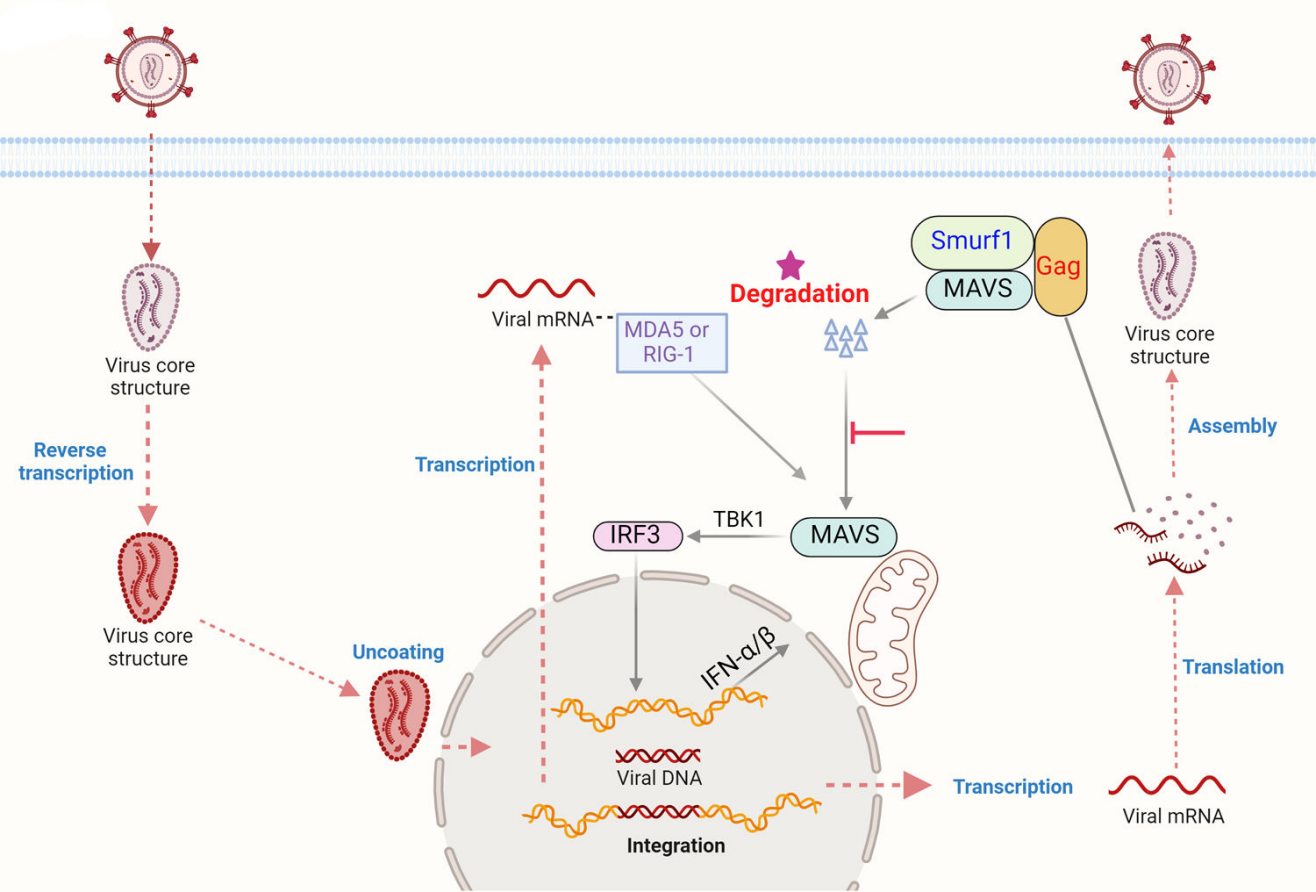
Schematic diagrams illustrate how EIAV negatively regulates RLR pathway activation by degrading MAVS. Virus infection results in activation of the RLR pathway and production of type I IFN. To escape such innate immunity of the host, in the late period of infection, EIAV core structure protein Gag binds to MAVS to recruit E3 ubiquitin ligase Smurf1, which can catalyze their K48-linked polyubiquitination for proteasome-dependent degradation. All above processes lead to the inhibition of the RLR signaling pathway and downregulation of IFN-β.

The *gag* and *pol* gene products of EIAV are translated from the full-length viral messenger RNA (41). Synthesis of Gag-Pol polyproteins requires ribosomes to shift their translational reading frame to read through the stop codon in *gag* (42). The assembly of Gag precursor proteins on the plasma membrane is essential for virus budding from the host cells. At the late stage of viral replication, during or shortly after virus budding from the host cell, the EIAV protease (PR) cleaves Pr55Gag into the mature Gag proteins p15 matrix (MA), p26 capsid (CA), p11 nucleocapsid (NC), and p9. The proteolytic processing of Gag induces a major transformation in virion structure: MA remains associated with the inner face of the viral membrane, whereas CA condenses to form a shell around the viral RNA/NC complex. This rearrangement, known as maturation, produces a morphological transition to a particle with a conical core characteristic of an infectious virion (43). Furthermore, about the viral invasion and replication process, there is increasing evidence that lentivirus capsids uncoated close to or at the nuclear pore after completion of reverse transcription (44), restricting release of MA and CA to cytoplasmic, so the conical capsid shell is mostly intact until it enters the nucleus. Consequently, there are a few free MA and CA in the cytoplasm after viral infection, and Pr55Gag proteins are produced after proviral DNA transcription. In our study, we found that the Gag’s domains p15 and p26, but not p11 and p9, target MAVS for degradation (Fig. 7B), and EIAV infection can induce high multiples of type I IFN at 48 hpi but inhibit it at 72 hpi (Fig. 1A). According to the principles of lentivirus replication, we speculated that after the virus has finished reverse transcription, replication, and translation, the Gag-precursor, but not the mature proteins p15 and p26, plays a key role in MAVS degradation (Fig. 10). To date, all studies conducted demonstrate extensive conservation of important structural and/or functional motifs of Gag, but the domains of Gag, especially p15 and p26, have very low homology and no common motif. We speculated that there were maybe some key amino acid residues of p15 or p26 that play key roles in their molecular interactions. To validate this hypothesis, we performed simulation analysis and coimmunoprecipitation, and identified the key sites involved in the interactions of p15 or p26 with MAVS or Smurf1 (Fig. 8 and 9).

We explored potential molecular mechanisms underlying MAVS degradation by EIAV Gag protein. The pharmacological experiments showed that EIAV Gag protein transfection degrades MAVS through the ubiquitin-proteasome pathway (Fig. 4A and B). Several E3 ligases have been identified as being involved in MAVS degradation to avoid excessive activation of IFN (45–47). To date, the E3 ligase AIP4, RNF5, and Smurf1 have been identified as being involved in virus-mediated MAVS degradation. As described above, SARS ORF-9b usurps AIP4 for the degradation of MAVS, and NDV V protein targets MAVS for ubiquitin-mediated degradation through E3 ubiquitin ligase RNF5 (11, 12). In addition, Sendai virus (SeV) infection promotes the deubiquitinase OTUD1 expression followed by upregulating protein levels of Smurf1 to degrade MAVS, and Smurf1 proteins interact with the MAVS/TRAF3/TRAF6 signalosome in cells infected with SeV for 8 h (48). This indicated that the function of some E3 ligases (e.g., Smurf1) to interact and degrade MAVS is enhanced in the early and middle stages of viral infection. In our experiment, we found the knockdown of Smurf1 could attenuate EIAV Gag protein-triggered MAVS degradation. Other E3 ligases, such as MUL1 or RNF5, also were involved in MAVS degradation (Fig. 4C). MUL1- or RNF5-mediated ubiquitination and degradation of MAVS are known to be mechanisms for the regulation of immune homeostasis (46, 47), and it might therefore be that, in the course of EIAV infection, MUL1 or RNF can degrade MAVS without dependence on virus or Gag proteins. Therefore, we further investigated which E3 ligases were usurped by EIAV to degrade MAVS to attenuate RLR pathway activation. The coimmunoprecipitation results showed that only Smurf1 was able to interact with Gag protein (Fig. 4D and E). Moreover, the accumulation and binding of Smurf1 protein with MAVS were promoted by Gag (Fig. 5). This indicated that EIAV Gag protein degrades mitochondrial antiviral signaling protein via E3 ubiquitin ligase Smurf1.

In summary, our work proposes a working model for uncovering mechanisms underlying EIAV Gag-mediated evasion of innate immunity: virus infection results in activation of the RLR pathway and the production of type I IFN. To escape such innate immunity of the host, EIAV Gag binds to MAVS and recruits the E3 ligase Smurf1, which can catalyze their K48-linked polyubiquitination of MAVS for proteasome-dependent degradation. Our findings therefore revealed a new mechanism of lentivirus-mediated evasion of the host’s innate immunity, which might provide a theoretical basis for clinical drug research and development.

## MATERIALS AND METHODS

### Cells and viruses

Preparations of eMDMs were obtained from equine PBMCs as described previously, with a minor modification (49). Briefly, PBMCs were isolated from 200 to 300 mL of heparinized horse peripheral blood by centrifugation through a HybriMax Histopaque cushion (density = 1.077 g/cm^3^; Sigma, USA). Isolated PBMCs were washed with RPMI 1640 medium (HyClone, USA) three times and were resuspended in RPMI 1640 medium supplemented with 10% horse serum (HyClone, USA). In addition, 10^4^ U/mL penicillin, 10^4^ µg/mL streptomycin, 2 mM L-glutamine, 0.1 mM nonessential amino acids, 1 mM sodium pyruvate, and 0.25 mM sodium HEPES, all of which were purchased from Gibco Corporation (USA), were added to the cultures. The cells were then seeded into tissue culture flasks (Corning, USA) at 5 × 10^6^ cells/25 cm^2^ and were incubated at 37°C in 5% CO_2_ for approximately 12 h. Nonadherent and loosely adherent cells were removed by mildly shaking the flasks before changing the medium, and the remaining adherent cells were further incubated for 3 d to allow differentiation into eMDMs. Human embryonic kidney (HEK293T) (ATCC CRL- 3216) cells were maintained in Dulbecco’s modified Eagle’s medium (HyClone, USA) with 10% fetal bovine serum (FBS) (Sigma, USA) and 1% penicillin and streptomycin (Gibco, USA), and were kept at 37°C in 5% CO_2_. NBL-6 cells (Horse Dermal Fibroblast Cells, ATCC CCL-57) were maintained in Dulbecco’s high-glucose modified Eagle’s medium (Sigma, USA) supplemented with 10% FBS (Sigma, USA) and 1% penicillin and streptomycin (Gibco, USA), and were kept at 37°C in 5% CO_2_.

The wild-type pathogenic EIAV strains isolated in China did not replicate well in cultivated cells, including primary host cells, such as donkey monocyte-derived macrophages (dMDMs). However, EIAV_DLV34_ is a dMDM-adapted EIAV strain that was derived from the pathogenic EIAV strain, EIAV_DV117_, by 34 passages in dMDMs, and replicates well in eMDMs. The rescued virus EIAV_CMV3-8_ was generated by transfecting the EIAV infectious clone pCMV3-8 into HEK293T cells (50).

### Reagents and antibodies

The proteasome inhibitor MG132 (C2211; Sigma-Aldrich, St. Louis, MO, USA) was used at 20 µM. Both autophagy inhibitors Wort (12–338; Sigma-Aldrich) and CQ (PHR1258; Sigma-Aldrich) were used at 20 µM. The mouse anti-actin (A1978), mouse anti-Flag (F1804), and mouse anti-HA (H9658) monoclonal antibodies; the rabbit anti-Flag (F7425) and the rabbit anti-HA (H6908) antibodies were purchased from Sigma-Aldrich. Rat anti-Myc (ab206486) antibody was purchased from Abcam (Cambridge, UK). Rabbit anti-GST (10000-0-AP) antibody was purchased from Proteintech (USA). DyLight 800-labeled goat anti-mouse (5230–0415) and DyLight 680-labeled goat anti-rabbit (5230–0403) secondary antibodies were purchased from KPL (USA). Alexa Fluor 488-conjugated goat anti-mouse antibody, Alexa Fluor 555-conjugated goat anti-rabbit antibody, and Alexa Fluor 647-conjuated rabbit anti-mouse antibody were purchased from Invitrogen (USA). Antibodies against eqMAVS, p26, and Gag were prepared in our laboratory.

### Plasmid construction, transfection, and immunoblotting

The equine MAVS (eqMAVS) and equine Smurf1 (eqSmurf1) genes used in this study were cloned from the cDNA of eMDMs using RT-PCR with the following set of primers: eqMAVS sense, 5′-ATGACGGTTGCCGAGGACAA-3′; eqMAVS anti-sense, 5′-CTGGAGCAGGCGCCTACGGT-3′; eqSmurf1 sense, 5′-ATGTCGAACCCCGGGACGCG-3′; eqSmurf1 anti-sense, 5′-CTCCACGGCAAAGCCACAGGT-3′. The eqMAVS constructs were obtained by cloning PCR-generated fragments into the pcDNA3.1 expression vector tagged with HA or Flag at the C-terminal. The eqSmurf1 expression vector was constructed by inserting eqSmurf1 gene into a pCAGGS vector with HA or Flag tag at the C-terminal. To construct pcDNA3.1-VN-eqMAVS-Flag and pcDNA3.1-VC-eqSmurf1-HA, gene fragments encoding the Venus residues 2 to 173 (VN) and Venus residues 154 to 238 (VC) were synthesized and cloned into the pcDNA3.1-eqMAVS-Flag and pcDNA3.1-eqSmurf1-HA vector. All expression vectors were generated using the In-Fusion cloning (Clontech, Felicia, CA, USA). The pCMV 3-8 vector is an infectious EIAV clone derived from cell-adapted EIAV_FDDV_ by replacing the U3 region of its long terminal repeat (LTR) with the cytomegalovirus (CMV) promoter. This clone was constructed as previously described and kept in our laboratory (50). Genes encoding viral structural (Env) and non-structural proteins (Tat and S2) were amplified from pCMV 3-8 and cloned into the pcDNA3.1-HA. Gag was amplified from pCMV 3-8 and cloned into the VR1012. The pcDNA3.1-Rev-HA was maintained in our laboratory and expressed EIAV Rev. pRK5-HA-ubiquitin-K63 (17606) and pRK5-HA-ubiquitin-K48 (17605) were obtained from Addgene (USA). RNF5, MARCH5, AIP4, MUL1, and RNF125 gene fragments were amplified from cDNA made from NBL-6 cells and then cloned into plasmid pcDNA3.1-HA to generate HA-tagged proteins. A series of deletion mutants of Gag, eqMAVS, and eqSmurf1 were generated using standard oligo-directed mutagenesis techniques.

HEK293T cells or NBL-6 cells were cultured in 6-well plates and transiently transfected with the indicated plasmids using PolyJet DNA reagent (SignaGen Laboratories, SL100688), following the manufacturer’s instructions. At 48 hpt, the cells were harvested, lysed in lysis buffer (50 mM HEPES-NaOH, pH 7.9, 100 mM NaCl, 50 mM KCl, 0.25% NP-40, 1 mM dithiothreitol), and then centrifuged at 10,000 × *g* for 5 min to remove the cell nuclei. The proteins in the cell lysates were separated using SDS-PAGE and then transferred onto nitrocellulose membranes (Millipore, USA). The membranes were blocked with 5% fat-free dry milk (BD, USA) in Tris-buffered saline (20 mM Tris-HCl, 150 mM NaCl) for 2 h at room temperature and then incubated with the indicated primary and secondary antibodies. Bands were analyzed by scanning blots using the Odyssey Imaging System (Li-Cor, Lincoln, NE, USA).

### Coimmunoprecipitation and ubiquitination assay

HEK293T cells were lysed with a cell lysis buffer (50 mM HEPES-NaOH, pH 7.9, 100 mM NaCl, 50 mM KCl, 0.25% NP-40, 1 mM dithiothreitol) containing 1 mM phenylmethylsulfonyl fluoride and protease inhibitors (Merck-Millipore). Lysates were centrifuged at 12,000 × *g* for 10 min and precipitated with anti-Flag, in conjunction with anti-Flag magnetic beads (Thermo Fisher Scientific), overnight at 4°C. The beads were washed with lysis buffer four times and eluted with SDS loading buffer by boiling for 10 min. Cell lysates (80 µL) were eluted with SDS loading buffer and boiled for 10 min. Proteins isolated from the beads and the cell lysates were subjected to immunoblot analysis. For *in vitro* ubiquitination (Ub) experiments, HEK293T cells were transfected with the indicated plasmids for 24 h, including the plasmids expressing Gag-Myc, MAVS-Flag, Ub-HA, Ub (K48), and Ub (K63). Cells were lysed under denaturing conditions in an SDS buffer (50 mM Tris-HCl, pH 7.5, 0.5 mM EDTA, 1 mM DTT, 1% SDS) by boiling for 10 min. The lysate was subjected to immunoprecipitation using anti-Flag magnetic beads and subsequent SDS-PAGE and immunoblotting. Ubiquitylated MAVS was detected with anti-HA antibody.

### Confocal microscopy

HEK293T cells were transfected with the indicated vectors using PolyJet DNA reagent, and eMDMs were infected with EIAV_CMV3-8_. At different time points, the cells were fixed with 4% paraformaldehyde for 30 min, permeabilized with 0.1% Triton X-100 for 15 min at room temperature, and blocked in 5% fat-free milk in PBS for 1 h. For the immunofluorescence assay, HEK293T cells were incubated with primary antibodies against HA or Flag tags at a 1:500 dilution in blocking solution for 2 h, and eMDMs were incubated with primary antibodies against eqMAVS or EIAV Gag. Then, the cells were stained using Alexa Fluor 488-conjugated goat anti-mouse antibody (Invitrogen, USA) or Alexa Fluor 555-conjugated goat anti-rabbit antibody (Invitrogen, USA) at a 1:500 dilution for 1 h. Finally, the nuclei were stained with DAPI (Beyotime, China) for 5 min, and cells were observed and imaged on a confocal microscope (LSM 880; Zeiss, Germany).

### RNA interference

Equine MAVS siRNA, Smurf1 siRNA, RNF5 siRNA, MARCH5 siRNA, AIP4 siRNA, MUL1 siRNA, RNF125 siRNA, and control scrambled siRNA were designed and synthesized by Sangon Biotech (China). The sequences targeting each gene were as follows: MAVS siRNA (GAGAUUCUGCCUUACUUGUTT), Smurf1 siRNA (ACUUGUCUUAUUUCCACUUTT), RNF125 siRNA (CCGUGUGCCUUGAGGUGUUTT), RNF5 siRNA (GUGUCCAGUAUGUAAAGCUTT), MARCH5 siRNA (GGGUGGAAUUGCGUUUGUUTT), MUL1 siRNA (UGUGCGGUCUGUUAAAGAATT), and AIP4 siRNA (AGUUGGACUCAAGGAUUUATT). 2.0 × 10^5^ eMDMs or HEK293T cells were seeded into 6-well plates and cultivated for 24 h, and then transfected with MAVS or Smurf1 siRNA or siRNAs of other genes using Lipofectamine RNAiMAX (Invitrogen, USA). Briefly, 100 pmol siRNA in 100 µL serum-free Opti-MEM medium (Gibco, USA) and 6 µL Lipofectamine RNAiMAX in 100 µL of Opti-MEM were mixed and incubated for 5 min at room temperature. The mixtures were then added dropwise to each well. The knockdown efficiency was determined at 24 hpt using quantitative real-time PCR analysis.

### Quantitative real-time PCR

Total RNA was extracted from eMDMs using a RNeasy mini kit (Qiagen, Germany) according to the manufacturer’s instructions, and then subjected to reverse transcription using Prime-Script RT reagent kit with a gDNA Eraser (Takara, Japan). The expression levels of equine IFN-β mRNA were quantified using SYBR-Green (Takara, Japan)-based real-time quantitative PCR analysis on an Agilent Mx3005P, according to the manufacturer’s protocols. Real-time RT-PCR was performed using the IFN-β primers: IFN-β-forward (5ʹGTGTTTCTCCACCACGGCTCTTT3ʹ) and IFN-β-reverse (5ʹGACCAATGCAGCATCCTCCTTCT3ʹ). ACTB was used as a housekeeping control to normalize the number of living cells. The ACTB primers were ACTB-forward (5ʹCATCTGCTGGAAGGTGGACAA3ʹ) and ACTB-reverse (5ʹCGACATCCGTAAGGACCTGTA3ʹ). Relative fold changes in gene expression were determined using the 2^-ΔΔCT^ threshold cycle (CT) method (51).

### Proximity ligation assay (PLA)

A PLA was performed to detect protein–protein interactions using fluorescence microscopy as previously described (52, 53). Briefly, HKE293T cells were cultured in 10-chamber microscopic slides and then co-transfected with plasmids Gag-Flag and MAVS-HA. Twenty-four hours later, the cells were fixed with 4% paraformaldehyde for 15 min and blocked with Duo-Link blocking buffer for 1 h at 37°C. Cells were then incubated with rabbit anti-HA and mouse anti-Flag monoclonal antibodies diluted with the specific DuoLink antibody diluents for 2 h, washed for 2 min in 1× wash buffer A, and further incubated for 1 h at 37°C with specific PLA probes under hybridization conditions. A ligase was then added to the cells for 30 min at 37°C, to form a concatemeric product extending from the oligonucleotide arm of the PLA probe. Fluorescence image acquisition was performed with confocal laser scanning microscopy (LSM880; Zeiss). The PLA dot was visualized as being distinctly fluorescent in the Texas red channel. The PLA signals were quantified using the BlobFinder software (Olink Biosciences, Sweden) (54).

### Molecular simulation

The structural data of p15, p26, MAVS, and Smurf1 were downloaded from Protein Data Bank and AlphaFold (1HEK, 1EIA, 4O9L, and F6R1Z0, respectively). Models of the complexes of p15 or p26 and MAVS, and p15 or p26 and Smurf1, were generated using the structural data on the ZDOCK Server (https://zdock.umassmed.edu/). The results were presented in the software PyMOL 2.5.

### Statistical analysis

All graphs presented in this study were created in GraphPad Prism version 6.0 (La Jolla, CA, USA). The statistical values were calculated using one-way analysis of variance (ANOVA) or two-tailed Student’s *t*-tests. Each data bar represents the mean value ± SEM (standard error of mean) of at least three independent experiments in all cases. Asterisks indicate the statistical significance: NS, no significance; *P < 0.05; **P < 0.01; ***P < 0.001.

## Data Availability

All data supporting the findings of this study are available within the article.

## SUPPLEMENTAL MATERIAL

Figure S1 jvi.01691-24-s0001.tif Analysis of the interaction between p9 and MAVS or Smurf1.

Supplemental legend jvi.01691-24-s0002.docx Legend for Fig. S1.

ASM does not own the copyrights to Supplemental Material that may be linked to, or accessed through, an article. The authors have granted ASM a non-exclusive, world-wide license to publish the Supplemental Material files. Please contact the corresponding author directly for reuse.
