# Supplementary figures and images for: Equine lentivirus Gag protein degrades mitochondrial antiviral signaling protein via the E3 ubiquitin ligase Smurf1
Source: J Virol. 2024 Dec 12;99(1):e01691-24. doi: 10.1128/jvi.01691-24 (PMC11784353; doi:10.1128/jvi.01691-24)

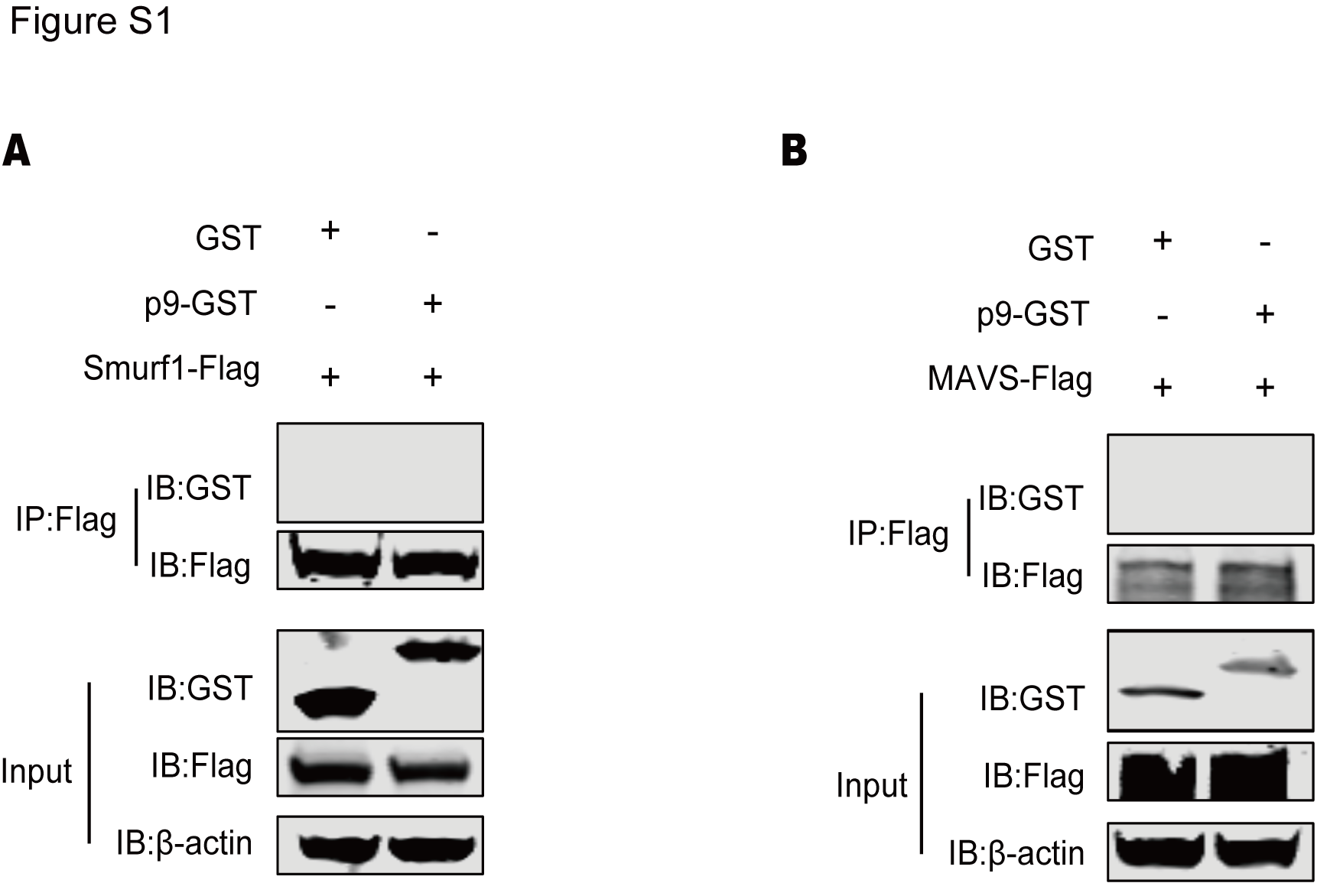

Supplement: Figure S1 — Analysis of the interaction between p9 and MAVS or Smurf1. [file jvi.01691-24-s0001.tif]
