# Supplementary material for: Equine lentivirus Gag protein degrades mitochondrial antiviral signaling protein via the E3 ubiquitin ligase Smurf1
Source: J Virol. 2024 Dec 12;99(1):e01691-24. doi: 10.1128/jvi.01691-24 (PMC11784353; doi:10.1128/jvi.01691-24)
Supplement: Supplemental legend — Legend for Fig. S1. [file jvi.01691-24-s0002.docx]

**Figure S1.** **Analysis of the interaction between p9 and MAVS or Smurf1.** (A) HEK-293T cells were co-transfected with MAVS-Flag and either empty vector or p9-GST. At 24 hpt, cells were harvested, immunoprecipitated with anti-Flag antibody, and further detected using immunoblot analysis with anti-Flag antibody. Expression levels of the proteins were analyzed by immunoblot analysis of the lysates with anti-GST, anti-Flag, or anti-β-actin antibody. The experiments were performed three times. (B) HEK293T cells were co-transfected with Smurf1-Flag and either empty vector or p9-GST. At 24 hpt, cells were harvested, immunoprecipitated with anti-Flag antibody, and further detected using immunoblot analysis with anti-Flag antibody. Expression levels of the proteins were analyzed by immunoblot analysis of the lysates with anti-GST, anti-Flag, or anti-β-actin antibody. The experiments were performed three times.
